# The nature and magnitude of cognitive impairment in narcolepsy type 1, narcolepsy type 2, and idiopathic hypersomnia: a meta-analysis

**DOI:** 10.1093/sleepadvances/zpae043

**Published:** 2024-06-26

**Authors:** Brian T Harel, James J Gattuso, Robert D Latzman, Paul Maruff, Thomas E Scammell, Giuseppe Plazzi

**Affiliations:** Neuroscience Therapeutic Area Unit, Takeda Development Center Americas, Inc., Cambridge, MA, USA; Florey Department of Neuroscience and Mental Health, University of Melbourne, Parkville, VIC, Australia; Neuroscience Therapeutic Area Unit, Takeda Development Center Americas, Inc., Cambridge, MA, USA; Cogstate Ltd, Melbourne, VIC, Australia; Department of Neurology, Beth Israel Deaconess Medical Center, Boston, MA, USA; IRCCS-Institute of Neurological Sciences, Bologna, Italy; Department of Biomedical, Metabolic and Neural Sciences, University of Modena and Reggio Emilia, Modena, Italy

**Keywords:** narcolepsy, idiopathic hypersomnia, cognitive impairment, sustained attention, meta-analysis

## Abstract

People with narcolepsy type 1 (NT1), narcolepsy type 2 (NT2), and idiopathic hypersomnia (IH) often report cognitive impairment which can be quite burdensome but is rarely evaluated in routine clinical practice. In this systematic review and meta-analysis, we assessed the nature and magnitude of cognitive impairment in NT1, NT2, and IH in studies conducted from January 2000 to October 2022. We classified cognitive tests assessing memory, executive function, and attention by cognitive domain. Between-group differences were analyzed as standardized mean differences (Cohen’s *d*), and Cohen’s *d* for individual tests were integrated according to cognitive domain and clinical disease group. Eighty-seven studies were screened for inclusion; 39 satisfied inclusion criteria, yielding 73 comparisons (*k*): NT1, *k* = 60; NT2, *k* = 8; IH, *k* = 5. Attention showed large impairment in people with NT1 (*d* = −0.90) and IH (*d* = −0.97), and moderate impairment in NT2 (*d* = −0.60). Executive function was moderately impaired in NT1 (*d* = −0.30) and NT2 (*d* = −0.38), and memory showed small impairments in NT1 (*d* = −0.33). A secondary meta-analysis identified sustained attention as the most impaired domain in NT1, NT2, and IH (*d ≈* −0.5 to −1). These meta-analyses confirm that cognitive impairments are present in NT1, NT2, and IH, and provide quantitative confirmation of reports of cognitive difficulties made by patients and clinicians. These findings provide a basis for the future design of studies to determine whether cognitive impairments can improve with pharmacologic and nonpharmacologic treatments for narcolepsy and IH.

## Graphical Abstract

**Graphical Abstract:**
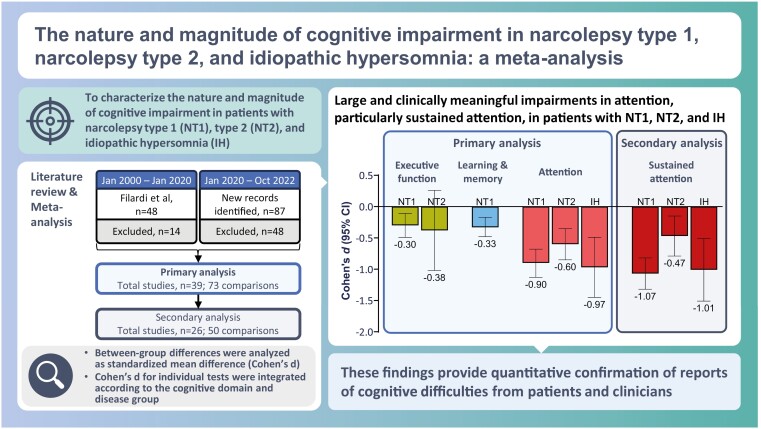

**Statement of Significance**

These meta-analyses confirm that cognitive impairment is an important symptom of narcolepsy type 1 (NT1), narcolepsy type 2 (NT2), and idiopathic hypersomnia (IH). Attention showed large impairment in people with NT1 and IH, and moderate impairment in NT2; memory showed small impairments in NT1. These patterns of cognitive impairment reveal the prominence of impairment in sustained attention across the three disorders.

Narcolepsy type 1 (NT1), narcolepsy type 2 (NT2), and idiopathic hypersomnia (IH) are rare central disorders of hypersomnolence characterized by excessive daytime sleepiness (EDS) arising from impaired central nervous system control of sleep–wake systems [1–5]. In addition, people with NT1 and NT2 often have disrupted nighttime sleep, sleep paralysis, and hypnogogic (falling to sleep) and hypnopompic (waking) hallucinations [2, 5–7]. NT1 is differentiated from NT2 by the presence of cataplexy (the sudden loss of muscle tone in response to strong emotions) and very low concentrations of orexin in cerebrospinal fluid [5]. In contrast, IH is characterized by EDS, sleep duration often >11 hours per day, and extreme sleep inertia [8]. All these disorders typically begin in adolescence or young adulthood.

There is now agreement from clinicians, expert scientific groups, and patients and their families that cognitive impairment is a common and disruptive symptom of NT1, NT2, and IH which is often unaddressed in clinics [2, 8–16]. Cognition encompasses all forms of thought, and theoretical models define specific types as well as component functions [17, 18]. For example, memory, executive function, and attention are distinct aspects of cognition, and within these, more specific functions can be defined and measured, such as reasoning, planning, and judgment (executive functions); focus, concentration, and sustained attention (attention); and long-term memory, learning and recognition memory (memory) [17].

In NT1, NT2, and IH, cognitive complaints are associated only weakly with performance on objective cognitive tests [11, 19], likely because subjective and objective cognitive assessments capture information about cognition differently. For example, a patient may complain they have difficulty remembering or solving problems, or they may describe general areas of difficulty using examples: a complaint about attention might be expressed as “difficulty staying on-task,” or a complaint about problem-solving expressed as “trouble doing sums or mental arithmetic.” Cognitive impairment may also be described in terms of problems with cognitively demanding daily activities; for example, “I have difficulty remembering the names of my students” or “I have trouble driving” [2, 8, 20, 21]. Depression or anxiety symptoms can also increase perceived cognitive difficulties, and, as a result, cognitive complaints are often associated with symptoms of depression or anxiety [19, 22].

The nature and magnitude of cognitive impairment in NT1, NT2, and IH have been examined with objective, validated tests of different cognitive functions [18]. Although models of impairment based on the objective assessment of cognition in NT1, NT2, and IH are still developing, some themes have emerged. First, most studies include only small samples (i.e. <30), most likely reflecting the rarity of these disorders [10]. Second, studies report impairment on cognitive tests only in the disorder they study [10]. Third, attentional function is assessed most often with impairment shown consistently on these tests [9, 11, 23–28]. Fourth, while impairment has been observed on tests of executive function and memory, these have been investigated much less frequently than attention [10]. Fifth, results of cognitive tests suggest impairment is more severe in NT1 than in NT2 [11, 29]. Together, these themes strongly suggest that cognition is impaired in NT1, NT2, and IH, but confirmation from systematic analyses and experimentation is required before they can be integrated into pathophysiological models of narcolepsy and IH.

The emphasis on impaired attention in NT1, NT2, and IH most likely reflects the broad impacts of poor attention [2, 6, 7]. One parsimonious model classifies attentional functions into four types [30]. First, sensory selective attention describes processes that moderate how information about external stimuli reaching sensory receptors is prioritized for processing, to ensure the organism can act in response to the most salient information [30, 31]. Second, controlled attention describes how attentional processes are allocated deliberately according to the characteristics of the information, the preparedness to respond, and the behavioral goal for which the information is to be used [30]. Third, focused attention describes how the processing of external or internal information is limited based on the complexity of that information, the behavioral goals of the organism, and the current levels of arousal and motivation [23, 30]. Fourth, sustained attention reflects the continuous application of these sensory selective, controlled, or focused attentional functions over time to influence ongoing interactions between individuals and their environments. This sustained activity can be influenced by physical factors such as levels of arousal and sleepiness and by psychological factors such as reward or reinforcement contingencies [30, 32, 33].

A recent descriptive review [10] sought to overcome the limitations associated with individual studies of small experimental samples, that have used many different cognitive tests, to develop a model of cognitive impairment in NT1, NT2, and IH. This was achieved by aggregating results from objective cognitive tests applied in peer-reviewed studies conducted between 2000 and 2020. Forty-eight unique, high-quality studies were identified [10], though 73% of studies were small (<30 patients). Although there was convergence in the cognitive functions measured, often the same function was assessed using different tests. To accommodate the small samples and test heterogeneity, data from different tests of the same cognitive function were aggregated across studies. Impaired performance on a test was classified if group mean performance in the NT1, NT2, or IH groups was arithmetically lower than in matched controls. For each cognitive function, the number of tests with impaired performance was summed for each patient group [10]. The review observed that most studies were conducted in NT1 (81.0%, *n* = 39), followed by NT2 (12.5%, *n* = 6), and IH (10.0%, *n* = 5), with more cognitive functions studied in NT1 (*n* = 19) than in NT2 (*n* = 7), and IH (*n* = 6). In NT1, impaired performance occurred most frequently for (1) attentional functions, such as alertness, selective attention, sustained attention, and vigilance, and (2) executive functions, such as decision-making under risk. In NT2, impaired performance occurred most frequently for sustained attention, working memory, set-shifting, and cognitive flexibility. In IH, impaired performance occurred most frequently for tests of alertness and concentration, although no other cognitive functions had been studied in this group. While these data confirmed that cognitive impairment occurred in NT1, NT2, and IH, particularly in sustained attention, simple counts of tests with lower performance in patent groups—ignoring the magnitude of differences, the associated group variability, sample sizes, and number of comparisons made—limits the generalizability of these results. Thus, while this descriptive analysis suggests that cognitive impairment does occur in NT1, NT2, and IH, theoretical and analytic models with greater precision and potential for generalization are needed to understand the importance of cognitive impairment for patients and integrate information about cognitive impairment into current pathophysiological models.

Pathophysiological models of medical disorders for which cognitive impairment is an important symptom can integrate this symptom and its relationship to brain function by organizing data from cognitive tests using a neuropsychological framework [17, 18, 28]. With test data organized this way, meta-analyses can provide reliable estimates of the nature and magnitude of impairment in different cognitive domains. Meta-analytic estimates of cognitive impairment, with their specification of the magnitude of impairment, associated confidence intervals, and statistical significance are superior to the counts of simple group differences conducted in previous reviews of NT1, NT2, and IH. Therefore, the aim of this study was to apply a neuropsychological framework and meta-analytic techniques to cognition studies of NT1, NT2, and IH. This will generate estimates of the nature and magnitude of cognitive impairment in each disorder and provide a statistically reliable and theoretically informed basis for generalizations about CNS function in these disorders, allowing for evaluation of the effects of pharmacotherapies on cognition.

## Materials and Methods

We conducted a systematic literature review and meta-analysis according to the Preferred Reporting Items for Systematic Reviews and Meta-Analyses (PRISMA) guidelines.

### Study inclusion–exclusion criteria

We included articles from January 2000 to January 2020 that were identified by Filardi et al. [10]; in addition, a separate PubMed search covering the period from January 2020 to October 2022 was completed using the same search strategy. An additional search was conducted using Google Scholar, and reference lists of the selected studies were reviewed. Studies eligible for inclusion in the meta-analysis were those that (1) contained at least one validated cognitive test that could be classified according to standard neuropsychological compendia for cognitive domains [17, 34]; (2) included a healthy control group; (3) provided sufficient information to compute a standardized effect size; and (4) included NT1, NT2, or IH clinical populations. Preclinical research papers, book chapters, reviews, thesis dissertations, case studies, and non–peer-reviewed material were excluded. A secondary review was conducted on those articles identified in the primary literature review to identify a subset of articles that included a neuropsychological test of attention.

### Review methodology

Two researchers (JJG and PM) reviewed the articles independently, with disagreements decided by two additional independent reviewers (BH and RDL). Data were then extracted by the original two researchers.

For each study identified for the primary meta-analysis, performance data from each cognitive test were organized in accordance with the cognitive function framework applied by Filardi et al. [10]. The test was then organized according to the main cognitive domain it assessed, defined according to standard neuropsychological frameworks [17, 18]. The identified tests and classification of neuropsychological domain are detailed in Supplementary Table S1. For the secondary meta-analysis, each test from the attention domain was reclassified according to the attentional function it measured, using a neuropsychological framework for attention (Supplementary Table S4) [30].

Publication bias was assessed using the trim-and-fill method and visual inspection of funnel plots [26].

### Statistical analysis

Statistical analyses were conducted using Comprehensive Meta-Analysis software, version 3.3 (Biostat, NJ, USA), and results are reported using a random-effects model [35]. Effect sizes (Cohen’s *d*) reflecting the standardized mean difference between groups for each cognitive test were calculated from the reported means and standard deviations of the NT1, NT2, or IH group studied and their matched control group. If study data did not contain the mean and standard deviation of the patient and control group but did provide estimates of standard error of the mean, geometrical means, medians, confidence intervals, interquartile ranges, sample size, correlation coefficient, and exact *p*-values, then effect sizes were computed independently using the procedures described in the Cochrane Handbook [36]. When studies reported summary statistics graphically, means and standard deviations were derived from the relevant figures. The signs of effect sizes for group differences were made uniform by reversing those (i.e. multiplied by −1) that reflected tests for which higher values indicated greater impairment (e.g. reaction times, number of errors). For studies that used more than one test to measure the same cognitive domain, effect sizes were averaged in that study before inclusion in the meta-analysis [37]. All group mean effect size estimates were weighted using inverse variance weighting based on sample size. Magnitudes of effect sizes were classified by convention [38] as small (0.2–0.49), medium (0.5–0.79), or large (0.8–1.2). Effect sizes <0.2 were classified as trivial and not interpreted, regardless of statistical significance.

The level of significance for heterogeneity tests in group means effect sizes was set to *p *< .1, as tests of heterogeneity often lack statistical power [39]. When statistically significant heterogeneity was identified, post hoc meta-regression analyses using the Knapp-Hartung method were conducted to seek the source [40, 41]. This analysis investigated the extent to which heterogeneity in the estimated mean effect arose from variance due to moderators such as (1) study location (Europe, Asia, America); (2) participant age; (3) number of cognitive measures used; (4) medication use (“yes” was reported if medication use at the time of the experiment occurred in ≥25% of participants); and (5) quality of the control group (recruited from community = 3, hospital or student = 2, other = 1, and undefined = 0, and declarations that a clinical sleep history or questionnaire was completed = 1, neurological examination was completed = 1, and assessment of central nervous system active medication was completed = 1, to provide a maximum score of 6 and minimum of 0). Following the Cochrane guidelines, moderator variables were only assessed if *k *≥ 10 [36]. Finally, we conducted sensitivity analyses investigating the impact on estimates of removing data from single studies for each cognitive domain to determine the reliability of results [35]. Statistical significance was set at *p < .*05 unless stipulated otherwise.

## Results

### Study identification

**Figure 1.**
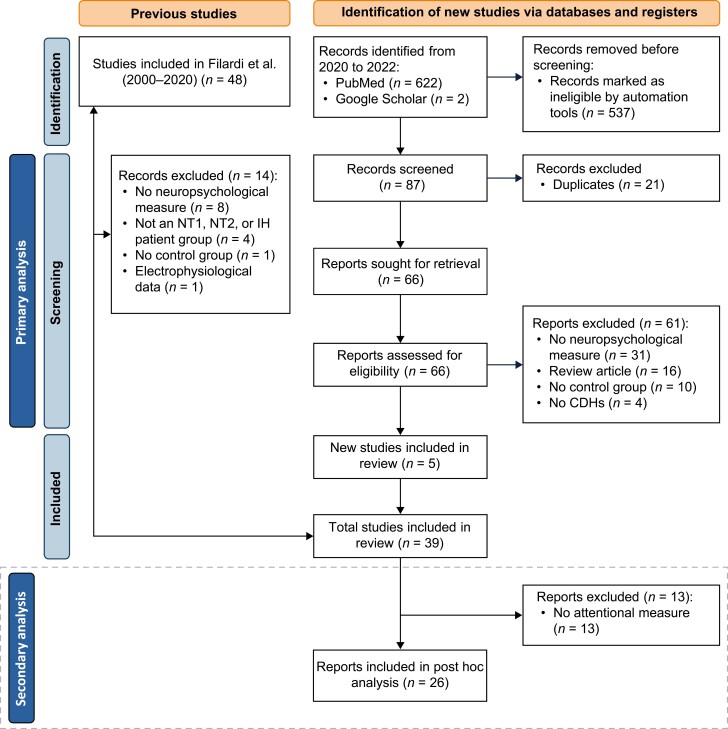
Preferred Reporting Items for Systematic Reviews and Meta-Analyses (PRISMA) flowchart for identification and integration of studies of cognitive function in narcolepsy type 1 (NT1), narcolepsy type 2 (NT2), and idiopathic hypersomnia (IH). The nodes shown in the box with the dotted outline pertain to the focused meta-analysis on specific attentional functions.

For the primary literature review, we reviewed all 48 studies from Filardi et al. [10], subsequently excluding 14 for further analysis on the application of the meta-analysis inclusion criteria for reasons described in Figure 1. Searches in PubMed and Google Scholar from January 2020 to October 2022 identified 624 studies, of which 21 were identified as duplicates and removed, and another 537 were excluded upon initial screening. The resulting 66 studies were subjected to the meta-analysis inclusion criteria, and 61 of those studies were excluded (Figure 1). This process resulted in the identification of five additional studies, for a total of 39 studies.

In the secondary review, the application of the additional inclusion criterion resulted in the exclusion of 13 studies from the initial set, providing a final total of 26 published studies (dotted box indicated in Figure 1). Reclassification of cognitive tests according to the attentional function they assessed resulted in the total sample size in the secondary meta-analysis becoming slightly different from that for tests classified as measuring the attention domain in the primary meta-analysis. This small difference arose because some individual studies (e.g. [16, 42],) included tests of different attentional functions. In the primary meta-analysis, these were combined at the study level while in the secondary meta-analyses, they were analyzed separately.

### Primary meta-analyses: impairment according to cognitive domain

For the primary meta-analyses of impairment according to the cognitive domain, we summarized the number of comparisons on the individual cognitive tests (*k*) and sample sizes for each disorder and their associated control group that provided the pooled effect size estimate for each cognitive domain (Table 1). Forest plots for effect sizes and post hoc sensitivity analyses are summarized in Supplementary Materials. Cognition has been studied most in NT1, with 60 comparisons (NT1, *n* = 1512; controls, *n* = 1454) providing estimates of performance in three cognitive domains. NT2 was studied less, with eight comparisons (NT2, *n* = 183; controls, *n* = 139) providing estimates for two cognitive domains. For IH, five group comparisons (IH, *n* = 186; controls, *n* = 155) provided estimates of performance for a single cognitive domain. Classification of tests according to cognitive domain stratification indicated that 33 comparisons were made of the attentional domain, 25 of the executive function domain, and 15 of the learning and memory domain. The negative signs for all pooled effect sizes indicated that for the cognitive domains assessed, performance was worse than in matched controls.

**Table 1.** Summary of Pooled Effect Sizes and Heterogeneity Statistics From the Meta-Analyses of Impairment in the Main Cognitive Domains in NT1, NT2, and IH

Cognitive domain	NT1		NT2		IH	
	*n, k*	Cohen’s *d* (95% CI)	*n, k*	Cohen’s *d* (95% CI)	*n, k*	Cohen’s *d* (95% CI)
Attention	NT1, *n* = 572 C, *n* = 541 *k* = 23	−0.90 (− 1.13 to −0.68)***^†^ *I*^*2*^ = 65.36	NT2, *n* = 117 C, *n* = 49 *k *= 5	−0.60 (−0.85 to −0.35)*** I^2^ = 0.00	IH, *n* = 186 C, *n* = 155 *k *= 5	−0.97 (−1.45 to −0.49)***^†^ *I*^2^ = 73.31
Executive function	NT1, *n* = 609 C, *n* = 574 *k *= 22	−0.30 (−0.49 to −0.11)**^†^*I*^*2*^* *= 59.30	NT2, *n* = 66 C, *n* = 90 *k *= 3	−0.38 (−1.02 to 0.26)^†^ *I*^*2*^* *= 73.20		
Learning and memory	NT1, *n* = 331 C, *n* = 339 *k *= 15	−0.33 (−0.48 to −0.17)*** I^*2*^* *= 0.00				

Cohen’s *d* represents the standardized difference in means between patients with NT1, NT2, or IH and their matched controls. An effect size with a negative sign indicates greater impairment in the patient group. Blank spaces indicate that there was zero or one study for the specific cognitive domain.

*p < .05; **p < .01; ***p < .001.

^†^Significant heterogeneity *p < *.10.

C, controls; CI, confidence interval; IH, idiopathic hypersomnia; NT1, narcolepsy type 1; NT2, narcolepsy type 2.

**Figure 2.**
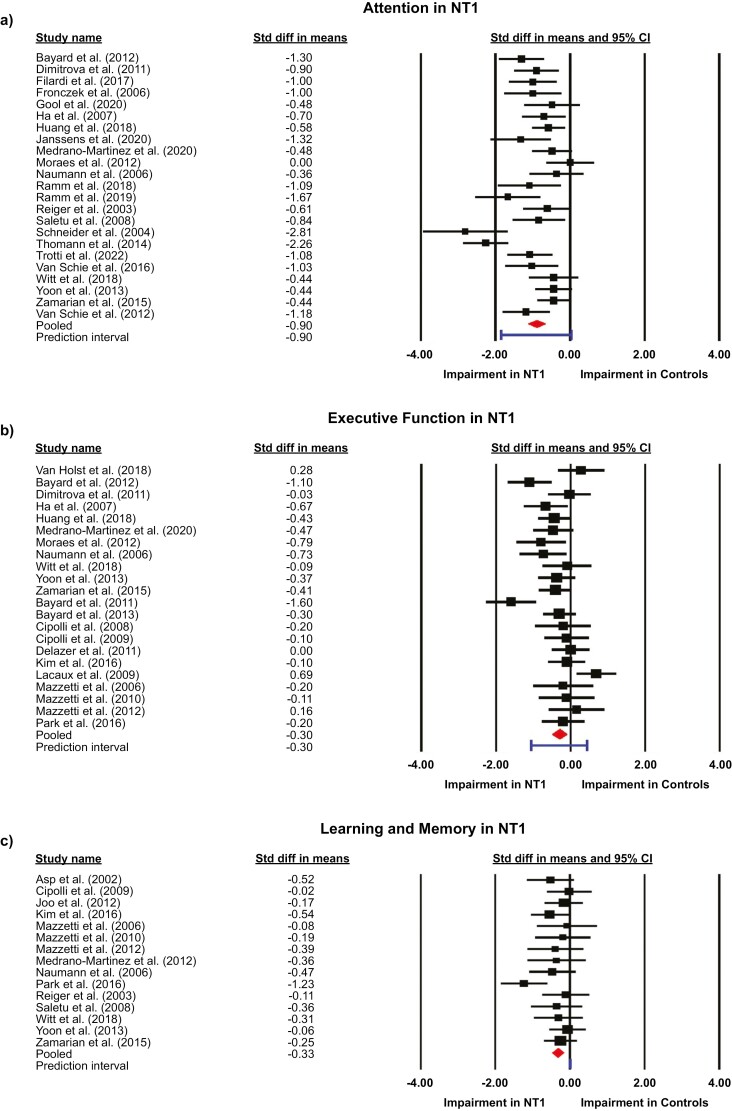
Individual and pooled effect sizes on neuropsychological tests of (a) attention, (b) executive function, and (c) learning and memory in narcolepsy type 1 (NT1). The sizes of the squares represent study weighting due to sample size. CI, confidence interval.

**Figure 3.**
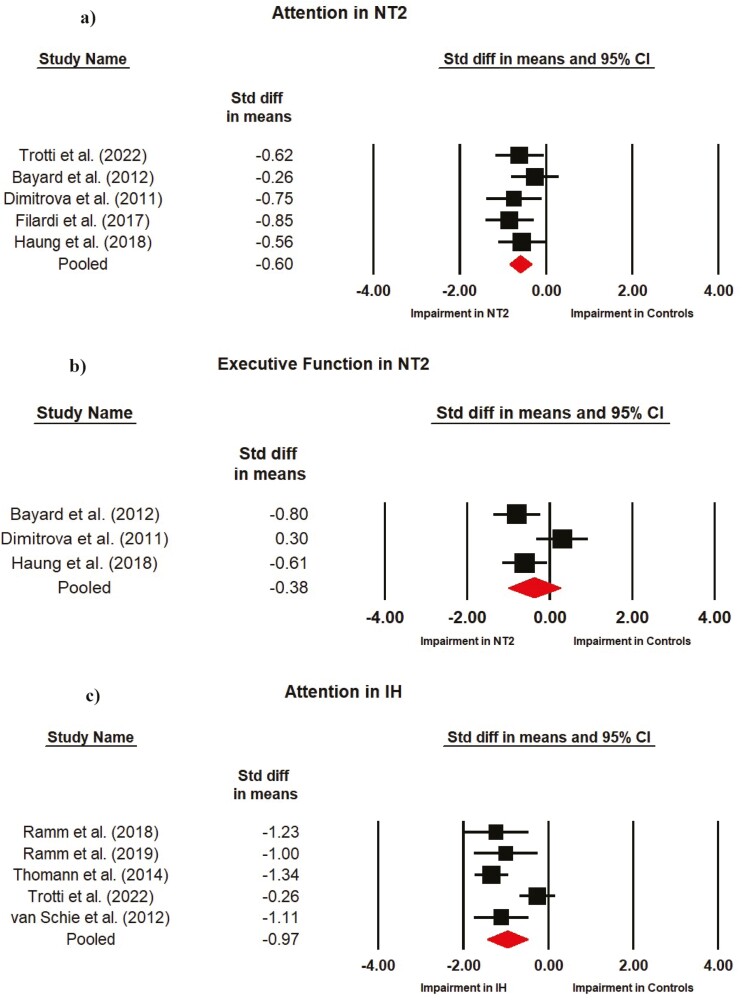
Individual and pooled effect sizes on neuropsychological tests of (a) attention and (b) executive function in narcolepsy type 2 (NT2) and of (c) attention in idiopathic hypersomnia (IH). The sizes of the squares represent study weighting due to sample size.

For NT1, there was a large impairment in attention that was statistically significant (*d* = −0.90; 95% CIs: −1.13 to −0.68). Impairments were small but statistically significant in executive function (*d* = −0.30; 95% CIs: −0.49 to −0.11) and learning and memory (*d* = −0.33; 95% CIs: −0.48 to −0.17). For NT2, attention showed a moderate impairment that was statistically significant (*d* = −0.60; 95% CIs: −0.85 to −0.35). For IH, attention showed a large impairment that was statistically significant (*d* = −0.91; 95% CIs: −1.45 to −0.49). In NT1, the contributions of the individual effect sizes to these pooled estimates are shown in Figure 2 for tests in attention (Figure 2a), executive function (Figure 2b), and learning and memory (Figure 2c). For NT2 the effect size of each study is shown for attention (Figure 3a) and executive function (Figure 3b) and in IH individual effect sizes and pooled estimates are shown for cognitive tests of attention (Figure 3c).

Post hoc sensitivity analyses indicated that for NT1, no removal of any one study significantly shifted the overall effect size for a cognitive domain (Supplementary Figure S1). However, for NT2 and IH, the removal of single studies did result in changes to effect sizes (Supplementary Figure S2), most likely owing to smaller sample sizes and fewer comparisons supporting these pooled outcomes.

Post hoc analyses of publication bias were conducted for each cognitive domain per group. For NT1 in each cognitive domain, the funnel plots indicated that data points were distributed symmetrically around the mean effect size, indicating no publication bias (Supplementary Figure S3). In NT2 there was no evidence of publication bias for the cognitive domains of attention (Supplementary Figure S4a) and executive function (Supplementary Figure S4b). For IH, there was evidence of publication bias for the cognitive domains of attention (Supplementary Figure S4c), suggesting that the mean effect size for attention in IH may have been inflated due to unpublished studies. Still, this interpretation should be considered with caution owing to the low number of comparisons.

Using meta-regression, we examined the extent to which attention for NT1 was influenced by patient age and sex, whether they were using medication at the time of assessment, whether they were from research sites in Europe, the United States, or Asia, the breadth of the neuropsychological tests applied, and the quality of the control group. We found no influence on meta-analytic estimates from any of these factors (Supplementary Table S2). In NT1, 18% of the variation in results in the executive function domain was accounted for by the quality of the control group, 17% was accounted for by the number of cognitive measures used by the study and 11% was explained the by average age of participants; however, none of these factors reached statistical significance (Supplementary Table S3). There were insufficient data to conduct meta-regression in NT2 or IH (*k *< 10 [36]).

### Secondary meta-analyses: impairment according to attentional function

For the secondary meta-analysis of impairment according to attentional function, we summarized the number of comparisons on the individual cognitive tests (*k*) and the sample sizes for each disorder and their associated control group that provided the pooled effect size estimate for each cognitive domain (Table 2). Forest plots for effect sizes and post hoc sensitivity analyses are summarized in Supplementary Figures S7–10. The NT1 group provided 37 different comparisons of 887 patients and 829 matched controls and allowed meta-analytic estimates for attentional functions. For NT2, there were six comparisons made of 160 patients and 149 controls, which allowed estimates of effect sizes for sensory selective and sustained attention elements. For IH, there were seven comparisons made of 212 patients and 195 controls, which provided sufficient data to estimate effect sizes for focused and sustained attention elements.

**Table 2.** Summary of Pooled Effect Sizes and Heterogeneity Statistics From the Meta-Analyses of Specific Attentional Functions in NT1, NT2, and IH

Attentional element	NT1		NT2		IH	
	*n, k*	Cohen’s *d* (95% CI)	*n, k*	Cohen’s *d* (95% CI)	*n, k*	Cohen’s *d* (95% CI)
Sensory selective attention	NT1, *n* = 94 C, *n* = 71 *k* = 4	−0.61 (−1.29 to −0.08)^†^ *I*^*2*^ = 77.04	NT2, *n* = 43 C, *n* = 54 *k *= 2	−0.34 (−0.74 to 0.07) *I*^2^ = 0		
Focused attention	NT1, *n* = 213 C, *n* = 222 *k *= 10	−0.59 (−0.98 to −0.2)**^†^ *I*^*2*^* *= 72.42			IH, *n* = 26 C, *n* = 40 *k *= 2	−0.71 (−1.74 to 0.31)^†^ *I*^2^ = 74.77
Controlled attention	NT1, *n* = 207 C, *n* = 193 *k *= 8	−0.37 (−0.62 to −0.12)** I^2^ = 33.48				
Sustained attention	NT1, *n* = 373 C, *n* = 343 *k *= 15	−1.07 (−1.32 to −0.82)***^†^ *I*^*2*^* *= 53.12	NT2, *n* = 117 C, *n* = 95 *k *= 4	−0.47 (−0.79 to −0.15)** I^2^ = 22.30	IH, *n* = 186 C, *n* = 155 *k *= 5	−1.01 (−1.51 to −0.51)***^†^ *I*^2^ = 74.89

Cohen’s *d* represents the standardized difference in means between patients with NT1 and healthy controls. A negative effect indicates greater impairment in the patient group. Blank spaces indicate that there was zero or one study for the specific cognitive domain.

*p < .05; **p < .01; ***p < .001.

^†^Significant heterogeneity *p < .*10.

C, controls; CI, confidence interval; IH, idiopathic hypersomnia; NT1, narcolepsy type 1; NT2, narcolepsy type 2.

Average effect sizes were negative for each type of attentional function analyzed for the NT1, NT2, and IH groups (Table 2). For NT1, there was a large and statistically significant impairment in sustained attention (*d *= −1.07; 95% CIs: −1.32 to −0.82) and small but statistically significant impairments in controlled attention (*d* = −0.37; 95% CIs: −0.98 to −0.20). For NT2, there was a moderate and statistically significant impairment in sustained attention (*d* = −0.47; 95% CIs: −0.79 to −0.15). For IH, there was a large and statistically significant impairment in sustained attention (*d* = −1.01; 95% CIs: −1.51 to −0.51). The contribution of the individual effect sizes to the pooled estimates of effect sizes are shown in the Forest plots for sustained attention (Supplementary Figure S5a), focused attention (Supplementary Figure S5b), sensory selective attention (Supplementary Figure S5c), and controlled attention (Supplementary Figure S5d) for the NT1 group; for sustained attention (Supplementary Figure S6a) and sensory selective attention (Supplementary Figure S6b) for the NT2 group; and for sustained attention (Supplementary Figure S6c) and focused attention (Supplementary Figure S6d) for the IH group.

Post hoc sensitivity analysis on estimates of pooled effect sizes for impairment in attentional functions in the NT1 group indicated that removal of any single study did not significantly alter the significance of the pooled effect size (Supplementary Figure S7). The removal of one study adjusted the statistical significance of the pooled effect size for sustained attention for NT2 and for focused attention for IH (Supplementary Figure S8). Analysis of publication bias indicated no effects for any attentional functions estimated in NT1 (Supplementary Figure S9), NT2 (Supplementary Figure S10a), or IH (Supplementary Figure S10b).

Post hoc analyses using a meta-regression showed no relationships between focused attention in NT1 or sustained attention in IH and factors such as patient age and sex, medication use at the time of assessment, continent of residence, the breadth of the neuropsychological tests applied, and the quality of the control group (Supplementary Tables S5 and S6).

## Discussion

This meta-analysis demonstrates that cognitive impairment is frequent in NT1, NT2, and IH, though these disorders differ in the nature and magnitude of impairment. The primary and secondary meta-analyses show that despite variability in the cognitive functions assessed in the published studies, statistically significant impairment was evident in at least one cognitive domain for each disorder (Table 1). Furthermore, for each disorder, the largest impairment detected was in the attention domain, and impairment was greater in NT1 and IH than in NT2.

### Cognitive impairment in NT1, NT2, and IH is considered in a neuropsychological framework

The reliable and large magnitude impairment in attention in NT1, NT2, and IH emphasizes that disruption to this cognitive domain is central to each disorder. This finding aligns with reports from patients and their families of their difficulties remaining alert, engaged, or focused [9, 19]. However, the available data limit the completeness of neuropsychological models of NT1, NT2, and IH. For example, compared to NT1, attentional impairment was less in NT2, while impairment in executive function was small in both disorders. In NT1, the impairment in learning and memory was similar in magnitude to that in executive function, though impairment in learning and memory could not be estimated in NT2. Differences in the magnitude of attentional impairment between NT1 and NT2 may reflect normal variability in the narcolepsy-related impairment as the sample sizes and number of comparisons providing estimates of attention impairment for NT2 were smaller than for NT1 (Table 2). However, as the magnitude of attentional impairment in each disorder was statistically significant, one hypothesis is that cognitive impairment in NT2 is qualitatively similar, but generally less severe than in NT1. Other researchers have also concluded that patients with NT2 show less cognitive impairment than with NT1, with the greater cognitive impairment in NT1 suggested to reflect a consequence of the greater orexin deficiency in this disorder [2, 5, 43]. This hypothesis could be challenged by determining whether the qualitative similarity between impairment in NT1 and NT2 extends to other cognitive domains, such as visual-spatial function or language, or whether patterns of impairment change will necessitate that information about these domains is added to models. As with NT2, data for cognitive tests from studies of IH were limited in number so reliable estimates of impairment could be developed only for attention (Table 2). The magnitude of impairment in attention in IH was similar to that in NT1, suggesting disruption in the same brain networks. This hypothesis could be tested in future studies by determining whether memory and executive function are also impaired less severely in IH.

The impairments identified here, particularly those in attention, provide a starting point for a detailed neuropsychological model, but additional data for other cognitive domains across NT1, NT2, and IH are required to increase the sophistication of such models. Consideration of the cognitive domains for which estimates of impairment are missing, or where small sizes limit their reliability, shows which cognitive domains need to be investigated further. Results from future studies can also be integrated into the neuropsychological framework developed here. Despite more work being required to complete neuropsychological profiles in NT1, NT2, and IH, the statistically reliable and large magnitude impairments observed in this study confirm the centrality of impairment in attention and the importance of cognitive symptoms generally. They provide a foundation for the integration of cognitive impairment into pathophysiological models of narcolepsy and IH.

### Impairment in attention in NT1, NT2, and IH

The overrepresentation of attention in cognitive studies in the literature allowed a secondary meta-analysis of impairment in specific attentional functions in NT1, NT2, and IH. For sustained attention, the results of this meta-analysis showed a large impairment in NT1 and IH and a moderate impairment in NT2. In NT1 there were also small to moderate impairments in focused and controlled attention. Thus, while the model of attentional impairment in NT1, NT2, and IH is incomplete, it does indicate unequivocally that the ability to sustain attention is impaired most in these disorders, albeit less severe in NT2. The results also indicate that, where they were measured, impairment in other attentional functions was smaller and more variable, though more data are required to obtain estimates of each attentional function in NT2 and IH. These results suggest that neuroscientific studies seeking to link impairment in cognition to brain-behavior networks in narcolepsy or IH should focus on brain networks known to be responsible for the ability to sustain attention over time.

### Cognitive impairment and sleepiness

One potential explanation for the finding of cognitive impairment in NT1, NT2, and IH is that the cognitive impairment detected may be an indirect consequence of the sleepiness that characterizes narcolepsy and IH [2, 44, 45]. This explanation is based on the premise that with increasing sleepiness there is a resultant reduction in the cognitive resources available to engage in cognitively demanding activities [9, 27, 45]. Thus, with more severe sleepiness, there should be greater cognitive impairment, and this should increase further with the difficulty of cognitive tests. However, results from individual studies examining this association are mixed, with cross-sectional studies finding weak associations between EDS symptoms and cognition [16, 24, 44, 46, 47], while others have failed to detect an association [9, 12, 13, 19, 42, 48]. One within-patient study reported that in NT1, NT2, and IH, cognitive impairment remains stable despite daily changes in EDS symptoms [42]. As noted previously [25], assessments of EDS vary across studies, potentially contributing to these mixed findings [49]. Relationships between EDS symptoms and cognition may become clearer by studying their covariation in the same individuals.

### Cognitive impairment and pathophysiological models of narcolepsy and IH

The consistent impairments in attention, particularly sustained attention, in NT1, NT2, and IH, coupled with less severe impairment in other areas of higher cognition in NT1, suggest strongly that dysfunction in brain networks involved in attention is central to these disorders. Orexin signaling promotes attention, likely via excitatory projections to the prefrontal cortex and other areas of the cortex [50], as well as via cholinergic, dopaminergic, and noradrenergic neurons [51]. Specifically, acetylcholine (ACh) neurons in the basal forebrain, dopamine (DA) neurons in the ventral tegmental area, and noradrenergic (NA) neurons in the locus coeruleus help promote attention, particularly in balancing reflexive processes with controlled or executive processes [52–55].

Considered from this perspective, the greater impairment in sustained attention in NT1, compared to NT2, raises the hypothesis that dysregulated orexin neurotransmission disrupts brain-behavior networks necessary for attentional processes [30]. In IH, the magnitude of impaired attention was equivalent to that in NT1, although it was not possible to determine whether impairment extended to other cognitive domains, and if so, what the magnitude of this was. As orexin levels are not deficient in IH, dysfunction of ACh, NE, or DA neurotransmission may arise from another cause. In healthy humans, antagonists to receptors for these neurotransmitters, given at doses that do not induce sleepiness, induce large-magnitude impairments in sustained attention [30, 56]. This same approach could be applied to understand the less severe impairment in attention and executive function observed for NT2.

### Clinical relevance of cognitive impairment in NT1, NT2, and IH

The magnitude of impairment of sustained attention in NT1, NT2, and IH can be considered clinically important for several reasons. First, patients with narcolepsy and IH report that cognitive difficulties are a serious problem that significantly impacts daily functioning and quality of life [22, 57]. It is worth noting that these reported difficulties often persist despite treatment [22, 58]. Second, there is considerable evidence across a range of diseases demonstrating the real-world significance of objectively assessed cognitive impairment. Poor cognition, as assessed by objective cognitive assessments, is associated with or predicts under-employment, impaired occupational/school functioning, reduced everyday functioning, inability to take part in leisure activities, and maintaining social relationships in healthy individuals [59–61] and in a variety of other disorders, including schizophrenia [62–65], depression [66, 67], bipolar disorder [68], cancer [69, 70], and multiple sclerosis [71, 72]. Third, cognitive impairments that are approximately one standard deviation below normative controls are considered by clinical neuropsychologists to warrant further investigation [17]. Fourth, in neuropsychiatric contexts, compendia of standardized diagnostic criteria recommend that cognitive impairment of at least one standard deviation less than matched controls is sufficient to warrant the classification of a minor neurocognitive disorder [28]. Once classified, clinicians should seek the cause of such an impairment and then work to manage the consequences of this for their patients. Fifth, cognitive impairment of the magnitude observed here for sustained attention can substantially diminish an individual’s ability to actively participate in work or study, manage their households and medical care, and meaningfully engage in social and cultural activities [73, 74]. Individuals with impaired sustained attention generally have difficulty remaining focused and engaged in demanding activities such as driving or in types of employment that emphasize speed and accuracy in relatively repetitive actions (such as customer service in busy stores, monitoring events or people in crowded environments, or maintaining engagement during an exam) [17, 73, 74].

### Future investigations of cognitive impairment in NT1, NT2, and IH

The outcomes of this meta-analysis support the inclusion of cognitive endpoints as outcomes in clinical studies of pharmacological treatments for NT1, NT2, or IH. These results can be used by researchers to identify cognitive domains in NT1, NT2, or IH for which there are missing data, and seek to understand whether, and by how much, cognition is impaired in those domains. Furthermore, the results of these meta-analyses provide reference data for future studies, whereby the magnitude of differences observed between patient groups and matched controls can be compared. Neuropsychological models could also be used to dissociate aspects of attentional processing to determine, with greater specificity, the nature of impairment in this aspect of cognition. Finally, though it remains unclear whether cognitive impairment in these disorders is independent of EDS, there is evidence to suggest they are distinct [9, 12, 16, 19, 24, 42, 44, 47, 48]. Studies using longitudinal designs to examine relationships between the time course of cognitive impairment and EDS, either in terms of diurnal variation or after pharmacological challenge, could provide a basis for understanding these relationships (e.g. Van Schie et al. [42]). This would provide greater precision than cross-sectional analytical approaches that merely seek to measure associations between the two types of symptoms in relatively small groups of patients with NT1, NT2, or IH.

### Conclusions

The neuropsychological framework and primary and secondary meta-analyses applied in this study clearly characterize and quantify the cognitive impairment in NT1, NT2, and IH. These results align with patient reports of cognitive difficulties and their impact on daily functioning and quality of life. Together, they suggest that cognitive impairment could be considered a core symptom in narcolepsy and IH and that evaluation of cognition in patients with narcolepsy or IH may provide clinicians with actionable information. In addition, the magnitude and pervasiveness of cognitive impairment in narcolepsy and IH also suggest that improving cognition should be a goal in developing treatments for these conditions.

## Supplementary Material

zpae043_suppl_Supplementary_Materials

## Data Availability

The datasets supporting this analysis will be made available from the corresponding author on reasonable request.

## References

[1] Sateia MJ. International classification of sleep disorders-third edition: highlights and modifications. Chest. 2014;146(5):1387–1394. doi: 10.1378/chest.14-0970 25367475

[2] Bassetti CLA , Adamantidis A, Burdakov D, et al. Narcolepsy - clinical spectrum, aetiopathophysiology, diagnosis and treatment. Nat Rev Neurol. 2019;15(9):519–539. doi: 10.1038/s41582-019-0226-9 31324898

[3] Heier MS , Evsiukova T, Wilson J, Abdelnoor M, Hublin C, Ervik S. Prevalence of narcolepsy with cataplexy in Norway. Acta Neurol Scand. 2009;120(4):276–280. doi: 10.1111/j.1600-0404.2009.01166.x 19456307

[4] Tió E , Gaig C, Giner-Soriano M, et al. The prevalence of narcolepsy in Catalunya (Spain). J Sleep Res. 2018;27(5):e12640. doi: 10.1111/jsr.12640 29171110

[5] Kornum BR , Knudsen S, Ollila HM, et al. Narcolepsy. Nat Rev Dis Primers. 2017;3:16100. doi: 10.1038/nrdp.2016.100 28179647

[6] Gool JK , Zhang Z, Oei M, et al. Data-driven phenotyping of central disorders of hypersomnolence with unsupervised clustering. Neurology. 2022;98(23):e2387–e2400. doi: 10.1212/WNL.0000000000200519 35437263 PMC9202524

[7] Lammers GJ , Bassetti CLA, Dolenc-Groselj L, et al. Diagnosis of central disorders of hypersomnolence: a reappraisal by European experts. Sleep Med Rev. 2020;52:101306. doi: 10.1016/j.smrv.2020.101306 32311642

[8] Billiard M , Sonka K. Idiopathic hypersomnia. Sleep Med Rev. 2016;29:23–33. doi: 10.1016/j.smrv.2015.08.007 26599679

[9] Naumann A , Bellebaum C, Daum I. Cognitive deficits in narcolepsy. J Sleep Res. 2006;15(3):329–338. doi: 10.1111/j.1365-2869.2006.00533.x 16911036

[10] Filardi M , D’Anselmo A, Agnoli S, et al. Cognitive dysfunction in central disorders of hypersomnolence: a systematic review. Sleep Med Rev. 2021;59:101510. doi: 10.1016/j.smrv.2021.101510 34166991

[11] Bayard S , Croisier Langenier M, Cochen De Cock V, Scholz S, Dauvilliers Y. Executive control of attention in narcolepsy. PLoS One. 2012;7(4):e33525. doi: 10.1371/journal.pone.0033525 22558075 PMC3338809

[12] Fronczek R , Middelkoop HA, van Dijk JG, Lammers GJ. Focusing on vigilance instead of sleepiness in the assessment of narcolepsy: high sensitivity of the Sustained Attention to Response Task (SART). Sleep. 2006;29(2):187–191. doi: 10.1093/sleep/29.2.187 16494086

[13] Uguccioni G , Lavault S, Chaumereuil C, Golmard JL, Gagnon JF, Arnulf I. Long-term cognitive impairment in Kleine-Levin syndrome. Sleep. 2016;39(2):429–438. doi: 10.5665/sleep.5458 26414895 PMC4712402

[14] Engström M , Vigren P, Karlsson T, Landtblom AM. Working memory in 8 Kleine-Levin syndrome patients: an fMRI study. Sleep. 2009;32(5):681–688. doi: 10.1093/sleep/32.5.681 19480235 PMC2675903

[15] Maski K , Trotti LM, Kotagal S, et al. Treatment of central disorders of hypersomnolence: an American Academy of Sleep Medicine clinical practice guideline. J Clin Sleep Med. 2021;17(9):1881–1893. doi: 10.5664/jcsm.9328 34743789 PMC8636351

[16] Ramm M , Jafarpour A, Boentert M, Lojewsky N, Young P, Heidbreder A. The Perception and Attention Functions test battery as a measure of neurocognitive impairment in patients with suspected central disorders of hypersomnolence. J Sleep Res. 2018;27(2):273–280. doi: 10.1111/jsr.12587 28771870

[17] Lezak MD , Howieson DB, Loring DW, Hannay HJ, Fischer JS. Neuropsychological assessment. 4th ed. New York, NY: Oxford University Press; 2004.

[18] Strauss E , Sherman EMS, Spreen O. A compendium of neuropsychological tests. administration, norms and commentary. 3rd ed. NY, NY: Oxford University Press; 2006.

[19] Zamarian L , Högl B, Delazer M, et al. Subjective deficits of attention, cognition and depression in patients with narcolepsy. Sleep Med. 2015;16(1):45–51. doi: 10.1016/j.sleep.2014.07.025 25434299

[20] Fulda S , Schulz H. Cognitive dysfunction in sleep disorders. Sleep Med Rev. 2001;5(6):423–445. doi: 10.1053/smrv.2001.0157 12531152

[21] Miglis MG , Guilleminault C. Kleine-Levin syndrome: a review. Nat Sci Sleep. 2014;6:19–26. doi: 10.2147/NSS.S44750 24470783 PMC3901778

[22] Maski K , Steinhart E, Williams D, et al. Listening to the patient voice in narcolepsy: diagnostic delay, disease burden, and treatment efficacy. J Clin Sleep Med. 2017;13(3):419–425. doi: 10.5664/jcsm.6494 27923434 PMC5337589

[23] Neo G , Chua FK. Capturing focused attention. Percept Psychophys. 2006;68(8):1286–1296. doi: 10.3758/bf03193728 17378415

[24] Yoon SM , Joo EY, Kim JY, Hwang KJ, Hong SB. Is high IQ protective against cognitive dysfunction in narcoleptic patients? J Clin Neurol. 2013;9(2):118–124. doi: 10.3988/jcn.2013.9.2.118 23626650 PMC3633189

[25] Filardi M , Pizza F, Tonetti L, Antelmi E, Natale V, Plazzi G. Attention impairments and ADHD symptoms in adult narcoleptic patients with and without hypocretin deficiency. PLoS One. 2017;12(8):e0182085. doi : 10.1371/journal.pone.0182085 28763482 PMC5538711

[26] Duval S , Tweedle R. A nonparametric “trim and fill” method for accounting for publication bias in meta-analysis. J Am Stat Assoc. 2000;95(449):89–98. doi: 10.1080/01621459.2000.10473905

[27] Witt ST , Drissi NM, Tapper S, et al. Evidence for cognitive resource imbalance in adolescents with narcolepsy. Brain Imaging Behav. 2018;12(2):411–424. doi: 10.1007/s11682-017-9706-y 28321606 PMC5880867

[28] American Psychiatric Association, American Psychiatric Association DSM-5 Task Force. Diagnostic and statistical manual of mental disorders: DSM-5. 5th ed. Arlington, VA: American Psychiatric Association; 2013.

[29] Huang YS , Hsiao IT, Liu FY, et al. Neurocognition, sleep, and PET findings in type 2 vs type 1 narcolepsy. Neurology. 2018;90(17):e1478–e1487. doi: 10.1212/WNL.0000000000005346 29602910

[30] Cohen RA. The neuropsychology of attention. 2nd ed. New York, NY: Springer; 2014.

[31] Pessoa L , Kastner S, Ungerleider LG. Neuroimaging studies of attention: from modulation of sensory processing to top-down control. J Neurosci. 2003;23(10):3990–3998. doi: 10.1523/JNEUROSCI.23-10-03990.2003 12764083 PMC6741071

[32] Esterman M , Rothlein D. Models of sustained attention. Curr Opin Psychol. 2019;29:174–180. doi: 10.1016/j.copsyc.2019.03.005 30986621

[33] Sarter M , Givens B, Bruno JP. The cognitive neuroscience of sustained attention: where top-down meets bottom-up. Brain Res Brain Res Rev. 2001;35(2):146–160. doi: 10.1016/s0165-0173(01)00044-3 11336780

[34] Sachdev PS , Blacker D, Blazer DG, et al. Classifying neurocognitive disorders: the DSM-5 approach. Nat Rev Neurol. 2014;10(11):634–642. doi: 10.1038/nrneurol.2014.181 25266297

[35] Borenstein M , Hedges LV, Higgins JPT, Rothstein HR. Introduction to meta-analysis. 1st ed. Hoboken, NJ: John Wiley & Sons, Ltd; 2009.

[36] Higgins JPT , Thomas J, Chandler J, et al. Cochrane handbook for systematic reviews of interventions. 2nd ed. Chichester, UK: John Wiley & Sons, Inc.; 2019.

[37] Baker JE , Lim YY, Pietrzak RH, et al. Cognitive impairment and decline in cognitively normal older adults with high amyloid-beta: a meta-analysis. Alzheimers Dement (Amst). 2017;6:108–121. doi: 10.1016/j.dadm.2016.09.002 28239636 PMC5315443

[38] Cohen J. A power primer. Psychol Bull. 1992;112(1):155–159. doi: 10.1037//0033-2909.112.1.155 19565683

[39] Fletcher J. What is heterogeneity and is it important? BMJ. 2007;334(7584):94–96. doi: 10.1136/bmj.39057.406644.68 17218716 PMC1767262

[40] Ellis PD. The essential guide to effect sizes. Cambridge, UK: Cambridge University Press; 2010.

[41] IntHout J , Ioannidis JP, Borm GF. The Hartung-Knapp-Sidik-Jonkman method for random effects meta-analysis is straightforward and considerably outperforms the standard DerSimonian-Laird method. BMC Med Res Methodol. 2014;14:25. doi: 10.1186/1471-2288-14-25 24548571 PMC4015721

[42] Van Schie MK , Thijs RD, Fronczek R, Middelkoop HA, Lammers GJ, Van Dijk JG. Sustained attention to response task (SART) shows impaired vigilance in a spectrum of disorders of excessive daytime sleepiness. J Sleep Res. 2012;21(4):390–395. doi: 10.1111/j.1365-2869.2011.00979.x 22098127

[43] Nishino S , Ripley B, Overeem S, Lammers GJ, Mignot E. Hypocretin (orexin) deficiency in human narcolepsy. Lancet. 2000;355(9197):39–40. doi: 10.1016/S0140-6736(99)05582-8 10615891

[44] Hood B , Bruck D. Sleepiness and performance in narcolepsy. J Sleep Res. 1996;5(2):128–134. doi: 10.1046/j.1365-2869.1996.00018.x 8795814

[45] Estabrooke IV , McCarthy MT, Ko E, et al. Fos expression in orexin neurons varies with behavioral state. J Neurosci. 2001;21(5):1656–1662. doi: 10.1523/JNEUROSCI.21-05-01656.2001 11222656 PMC6762959

[46] Moraes M , Rossini S, Reimao R. Executive attention and working memory in narcoleptic outpatients. Arq Neuropsiquiatr. 2012;70(5):335–340. doi: 10.1590/s0004-282x2012005000007 22323335

[47] Ramm M , Boentert M, Lojewsky N, Jafarpour A, Young P, Heidbreder A. Disease-specific attention impairment in disorders of chronic excessive daytime sleepiness. Sleep Med. 2019;53:133–140. doi: 10.1016/j.sleep.2018.09.021 30508781

[48] Delazer M , Hogl B, Zamarian L, et al. Executive functions, information sampling, and decision making in narcolepsy with cataplexy. Neuropsychology. 2011;25(4):477–487. doi: 10.1037/a0022357 21463040

[49] Alexandre C , Andermann ML, Scammell TE. Control of arousal by the orexin neurons. Curr Opin Neurobiol. 2013;23(5):752–759. doi: 10.1016/j.conb.2013.04.008 23683477 PMC3783629

[50] Peyron C , Tighe DK, van den Pol AN, et al. Neurons containing hypocretin (orexin) project to multiple neuronal systems. J Neurosci. 1998;18(23):9996–10015. doi: 10.1523/JNEUROSCI.18-23-09996.1998 9822755 PMC6793310

[51] Villano I , Messina A, Valenzano A, et al. Basal forebrain cholinergic system and orexin neurons: effects on attention. Front Behav Neurosci. 2017;11:10. doi: 10.3389/fnbeh.2017.00010 28197081 PMC5281635

[52] Mason ST. Noradrenaline and selective attention: a review of the model and the evidence. Life Sci. 1980;27(8):617–631. doi: 10.1016/0024-3205(80)90001-6 6997668

[53] Del Campo N , Chamberlain SR, Sahakian BJ, Robbins TW. The roles of dopamine and noradrenaline in the pathophysiology and treatment of attention-deficit/hyperactivity disorder. Biol Psychiatry. 2011;69(12):e145–e157. doi: 10.1016/j.biopsych.2011.02.036 21550021

[54] Klinkenberg I , Sambeth A, Blokland A. Acetylcholine and attention. Behav Brain Res. 2011;221(2):430–442. doi: 10.1016/j.bbr.2010.11.033 21108972

[55] Nieoullon A. Dopamine and the regulation of cognition and attention. Prog Neurobiol. 2002;67(1):53–83. doi: 10.1016/s0301-0082(02)00011-4 12126656

[56] Thiele A , Bellgrove MA. Neuromodulation of attention. Neuron. 2018;97(4):769–785. doi: 10.1016/j.neuron.2018.01.008 29470969 PMC6204752

[57] Stevens J , Schneider LD, Husain AM, et al. Impairment in functioning and quality of life in patients with idiopathic hypersomnia: the real world idiopathic hypersomnia outcomes study (ARISE). Nat Sci Sleep. 2023;15:593–606. doi: 10.2147/NSS.S396641 37551277 PMC10404411

[58] Trotti LM , Ong JC, Plante DT, Friederich Murray C, King R, Bliwise DL. Disease symptomatology and response to treatment in people with idiopathic hypersomnia: initial data from the Hypersomnia Foundation registry. Sleep Med. 2020;75:343–349. doi: 10.1016/j.sleep.2020.08.034 32950878 PMC7669698

[59] Shibaoka M , Masuda M, Iwasawa S, Ikezawa S, Eguchi H, Nakagome K. Relationship between objective cognitive functioning and work performance among Japanese workers. J Occup Health. 2023;65(1):e12385. doi: 10.1002/1348-9585.12385 36694368 PMC9874020

[60] Shaikh KT , Tatham EL, Vandermorris S, et al. The impact of memory change on everyday life among older adults: association with cognition and self-reported memory. J Int Neuropsychol Soc. 2021;27(9):896–904. doi: 10.1017/S1355617720001344 33441202

[61] Suchy Y , Gereau Mora M, DesRuisseaux LA, Brothers SL. It’s complicated: executive functioning moderates impacts of daily busyness on everyday functioning in community-dwelling older adults. J Int Neuropsychol Soc. 2023;29(9):850–858. doi: 10.1017/S1355617723000048 37057862

[62] Takeda T , Tomotake M, Ueoka Y, et al. Relationship between cognitive function and employment in Japanese schizophrenia patients. Open J Psychiatry. 2016;06(1):65–70. doi: 10.4236/ojpsych.2016.61007

[63] Fu S , Czajkowski N, Rund BR, Torgalsboen AK. The relationship between level of cognitive impairments and functional outcome trajectories in first-episode schizophrenia. Schizophr Res. 2017;190:144–149. doi: 10.1016/j.schres.2017.03.002 28302394

[64] Nuechterlein KH , Subotnik KL, Green MF, et al. Neurocognitive predictors of work outcome in recent-onset schizophrenia. Schizophr Bull. 2011;37(suppl 2):S33–S40. doi: 10.1093/schbul/sbr084 21860045 PMC3160123

[65] Tandberg M , Ueland T, Sundet K, et al. Neurocognition and occupational functioning in patients with first-episode psychosis: a 2-year follow-up study. Psychiatry Res. 2011;188(3):334–342. doi: 10.1016/j.psychres.2011.04.021 21575993

[66] Knight MJ , Air T, Baune BT. The role of cognitive impairment in psychosocial functioning in remitted depression. J Affect Disord. 2018;235:129–134. doi: 10.1016/j.jad.2018.04.051 29655074

[67] Woo YS , Rosenblat JD, Kakar R, Bahk WM, McIntyre RS. Cognitive deficits as a mediator of poor occupational function in remitted major depressive disorder patients. Clin Psychopharmacol Neurosci. 2016;14(1):1–16. doi: 10.9758/cpn.2016.14.1.1 26792035 PMC4730927

[68] Depp CA , Mausbach BT, Harmell AL, et al. Meta-analysis of the association between cognitive abilities and everyday functioning in bipolar disorder. Bipolar Disord. 2012;14(3):217–226. doi: 10.1111/j.1399-5618.2012.01011.x 22548895 PMC3396289

[69] Hou Y , Wu X, Allen T, et al. Longitudinal association between executive function and academic achievement in children with neurofibromatosis type 1 and plexiform neurofibromas. J Int Neuropsychol Soc. 2023;29(9):839–849. doi: 10.1017/S1355617723000103 36750981 PMC10695331

[70] Noll KR , Bradshaw ME, Weinberg JS, Wefel JS. Neurocognitive functioning is associated with functional independence in newly diagnosed patients with temporal lobe glioma. Neurooncol Pract. 2018;5(3):184–193. doi: 10.1093/nop/npx028 30094046 PMC6075221

[71] Morrow SA , Drake A, Zivadinov R, Munschauer F, Weinstock-Guttman B, Benedict RH. Predicting loss of employment over three years in multiple sclerosis: clinically meaningful cognitive decline. Clin Neuropsychol. 2010;24(7):1131–1145. doi: 10.1080/13854046.2010.511272 20830649

[72] Benedict RH , Wahlig E, Bakshi R, et al. Predicting quality of life in multiple sclerosis: accounting for physical disability, fatigue, cognition, mood disorder, personality, and behavior change. J Neurol Sci. 2005;231(1–2):29–34. doi: 10.1016/j.jns.2004.12.009 15792817

[73] Maruff P , Jaeger J. Understanding the importance of cognitive dysfunction and cognitive change in major depressive disorder. In: McIntyre RS, ed. Cognitive impairment in major depressive disorder. Clinical relevance, biological substrates, and treatment opportunities. Cambridge, UK: Cambridge University Press; 2016: 15–29.

[74] Marcotte TD , Scott JC, Kamat R, Heaton RK. Neuropsychology and the prediction of everyday functioning. In: Marcotte TD, Grant I, eds. Neuropsychology of everyday functioning. New York, NY: The Guildford Press; 2010: 5–38.

