# Supplementary material for: The nature and magnitude of cognitive impairment in narcolepsy type 1, narcolepsy type 2, and idiopathic hypersomnia: a meta-analysis
Source: Sleep Adv. 2024 Jun 26;5(1):zpae043. doi: 10.1093/sleepadvances/zpae043 (PMC11258808; doi:10.1093/sleepadvances/zpae043)
Supplement: zpae043_suppl_Supplementary_Materials [file zpae043_suppl_supplementary_materials.docx]

**Supplementary Materials**

**Table of Contents**

[Study 1. Meta-analyses of cognitive impairment in NT1, NT2, and IH 3](#_Toc158115148)

[1.1. Classification of cognitive tests to the main neuropsychological domain measured. 3](#_Toc158115149)

[**Supplementary Table S1.** Neuropsychological framework for organizing tests of cognitive functions according to their main cognitive domain 3](#_Toc158115150)

[1.2. Post hoc analyses of sensitivity of pooled estimates of impairment in cognitive domains to the influence of single studies and publication bias. 5](#_Toc158115154)

[**Supplementary Figure S1.** Sensitivity analysis for (a) attention, (b) executive function, and (c) learning and memory domains in narcolepsy type 1 (NT1). The sizes of the squares represent study weighting due to sample size. CI, confidence interval. 6](#_Toc158115155)

[**Supplementary Figure S2.** Sensitivity analysis for (a) attention and (b) executive function domains in narcolepsy type 2 (NT2) and for (c) the attention domain in idiopathic hypersomnia (IH). The sizes of the squares represent study weighting due to sample size. CI, confidence interval. 7](#_Toc158115156)

[**Supplementary Figure S3.** Funnel plots of standard error by standard deviation in means for narcolepsy type 1 for (a) attention, (b) executive function, and (c) learning and memory. 8](#_Toc158115157)

[**Supplementary Figure S4.** Funnel plots of standard error by standard deviation in means for narcolepsy type 2 (NT2) and idiopathic hypersomnia (IH) for (a) attention in NT2, (b) executive function in NT2, and (c) attention in IH. 9](#_Toc158115158)

[1.3. Post hoc analyses of the influence of study characteristics on pooled estimates of attention and executive function. 10](#_Toc158115159)

[**Supplementary Table S2.** Meta-regression examining the influence of moderator variables on attention in narcolepsy type 1 10](#_Toc158115160)

[**Supplementary Table S3.** Meta-regression examining the influence of moderator variables on executive function in idiopathic hypersomnia 11](#_Toc158115161)

[Study 2: Post hoc meta-analyses of cognitive impairment in NT1, NT2, and IH 12](#_Toc158115162)

[2.1. Classification of cognitive tests according to attentional functions. 12](#_Toc158115164)

[**Supplementary Table S4.** Summary of attentional tests used in studies of narcolepsy type 1, narcolepsy type 2, and idiopathic hypersomnia, classified according to the attentional subdomain assessed 12](#_Toc158115165)

[2.2. Contribution of data from individual tests to post hoc pooled estimates of impairment in each attention function determined from meta-analysis. 14](#_Toc158115166)

[**Supplementary Figure S5.** Individual and pooled effect sizes of neuropsychological tests of attention subdomains in narcolepsy type 1 (NT1). The sizes of the squares represent study weighting due to sample size. CI, confidence interval. 15](#_Toc158115167)

[**Supplementary Figure S6.** Individual and pooled effect sizes of neuropsychological tests of attention subdomains in narcolepsy type 2 (NT2) and idiopathic hypersomnia (IH). The sizes of the squares represent study weighting due to sample size. CI, confidence interval. 16](#_Toc158115168)

[2.3. Post hoc analyses of sensitivity of post hoc pooled estimates of impairment in attentional functions to the influence of single studies and publication bias. 17](#_Toc158115169)

[**Supplementary Figure S7.** Sensitivity analysis of attention subdomains in narcolepsy type 1 (NT1). CI, confidence interval. 18](#_Toc158115170)

[**Supplementary Figure S8.** Sensitivity analysis of attention subdomains in narcolepsy type 2 (NT2) and idiopathic hypersomnia (IH). CI, confidence interval. 20](#_Toc158115171)

[**Supplementary Figure S9.** Funnel plots for attentional subdomains in narcolepsy type 1: (a) sustained attention, (b) focused attention, (c) sensory selective attention, and (d) controlled attention. 22](#_Toc158115172)

[**Supplementary Figure S10.** Funnel plots of attentional subdomains in narcolepsy type 2 (NT2) and idiopathic hypersomnia (IH): (a) sustained attention in NT2 and (b) sustained attention in IH. 23](#_Toc158115173)

[**Supplementary Table S5.** Meta-regression examining the influence of moderator variables on focused attention in narcolepsy type 1 24](#_Toc158115174)

[**Supplementary Table S6.** Meta-regression examining the influence of moderator variables on sustained attention in idiopathic hypersomnia 25](#_Toc158115175)

[References 26](#_Toc158115176)

# Study 1. Meta-analyses of cognitive impairment in NT1, NT2, and IH

## Classification of cognitive tests to the main neuropsychological domain measured.

### **Supplementary Table S1.** Neuropsychological framework for organizing tests of cognitive functions according to their main cognitive domain

| **Cognitive domain** | **Neuropsychological test** |
| --- | --- |
| Attention | Simple Reaction Time Tasks [1-4]  Complex and Multiple Response Time Tasks [5]  Attention Network Test [6] Visual and Acoustic Parallel Tasks [1]  Single and Dual Visual and Auditory Tasks [7]  Divided Attention [3]  Selective Attention Task [3,8]  Visual Scanning Task [7]  Visual Tracking Test [9]  Psychomotor Vigilance Task [10-12]  Vigilance Task [2,4,8]  Continuous Performance [4,13]  Sustained Attention Task [3]  Sustained Attention to Response Task [12,14-17]  Bourdon Vos Task [17]  d2 Test [1,18]  Forward Digit Span [1,5,19,20] |
| Executive function | Go-no Go Task [18,21]  Stroop Task [5,22-24]  Trail-Making-Test A [5,20,23,25-27]  Digit Test (arithmetic) [5]  Digit Test (backward) [1,5,19]  Continuous Performance Perseverations Score [13]  Updating (2-back task) [1,2]  Corsi Block Tapping [4,19]  Working Memory Composite Score [23,28]  Letter-Number Sequencing [22]  Verbal Working Memory [18]  Work Performance Test [4]  Set-shifting [2,21]  Cognitrone [4]  One Touch Stockings of Cambridge [18]  Iowa Gambling Task [21,29,30]  Game of Dice Task [29,30]  Balloon Analogue Risk Task [31]  Executive Function Composite Score [28]  Hayling Sentence Completion Test [1]  Baddeley’s Logical Reasoning Test [32]  Trail-Making-Test B [5,19,20,22,23,25-27,32,33]  Wisconsin Card Sorting Test [13]  Information Sampling Task [21]  Verbal Fluency [1,5,18,23,28] |
| Learning and memory | Immediate recall [1]  Rey-Osterrieth Complex Figure Test [19,20,23,34]  Weschler Memory Scale [25-27,32]  Benton Visual Retention Test [1]  Rey Auditory Verbal Learning Test [7]  California Verbal Learning Test [23,28,34]  Delayed Recall [1]  Total Verbal Memory (Grünberger) [35]  Verbal Learning and Memory Test [18]  2D Memory Task [36] |

## Post hoc analyses of sensitivity of pooled estimates of impairment in cognitive domains to the influence of single studies and publication bias.


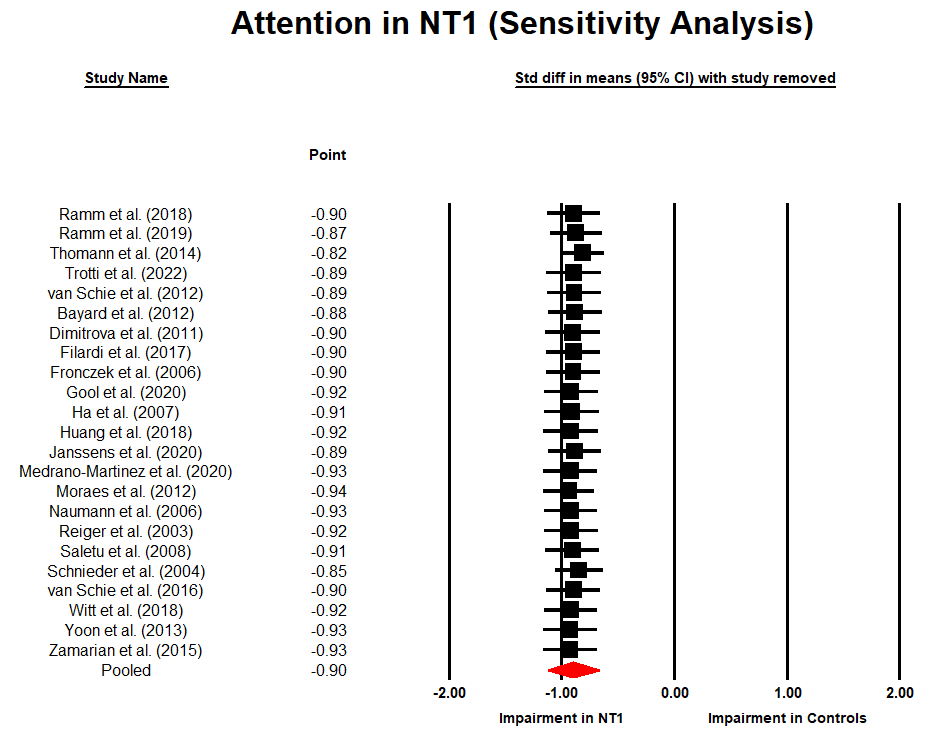


**a)**


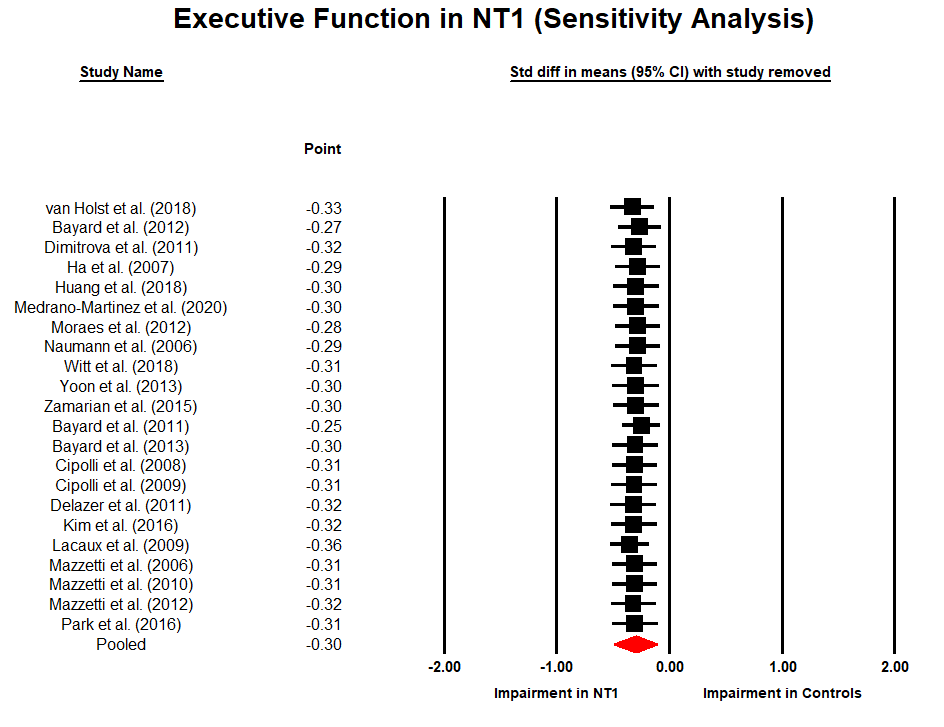


**b)**

**
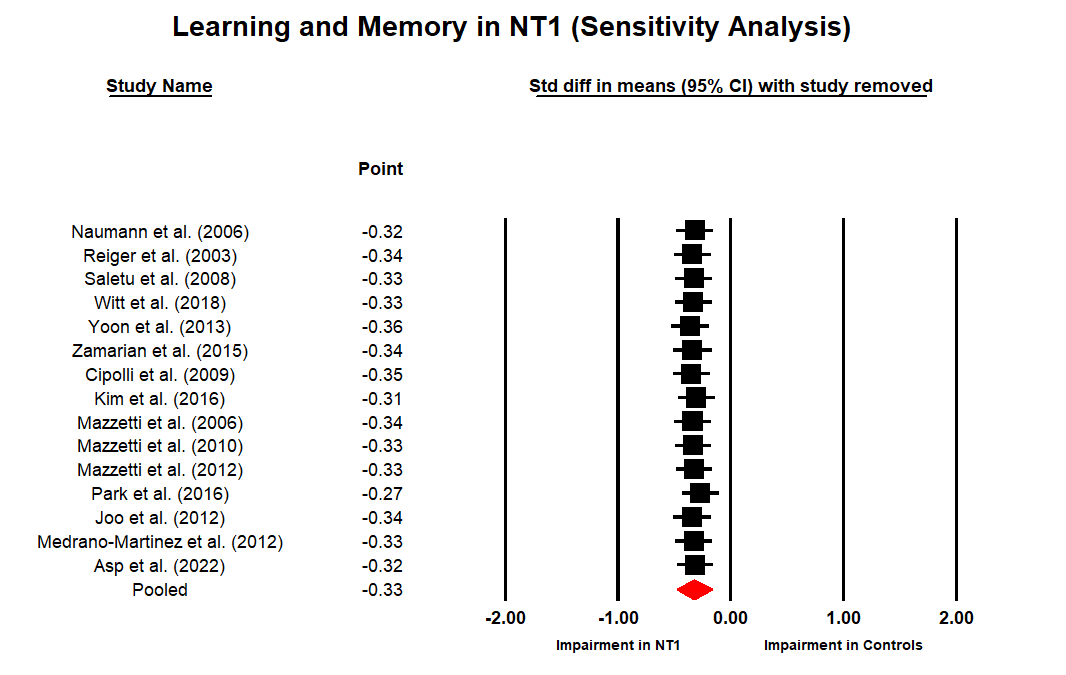
**

**c)**

### **Supplementary Figure S1.** Sensitivity analysis for (a) attention, (b) executive function, and (c) learning and memory domains in narcolepsy type 1 (NT1). The sizes of the squares represent study weighting due to sample size. CI, confidence interval.


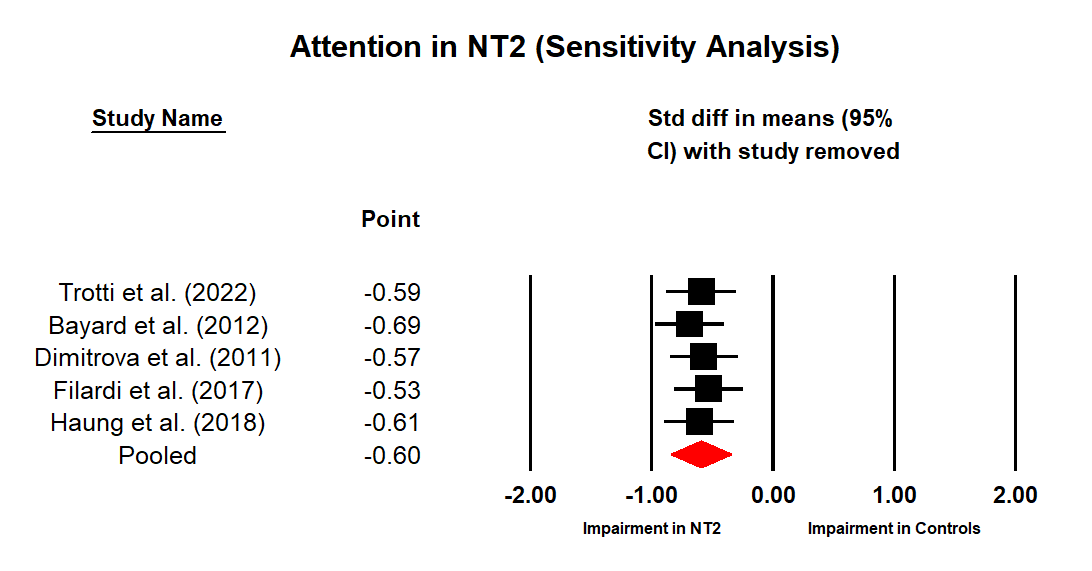


**a)**


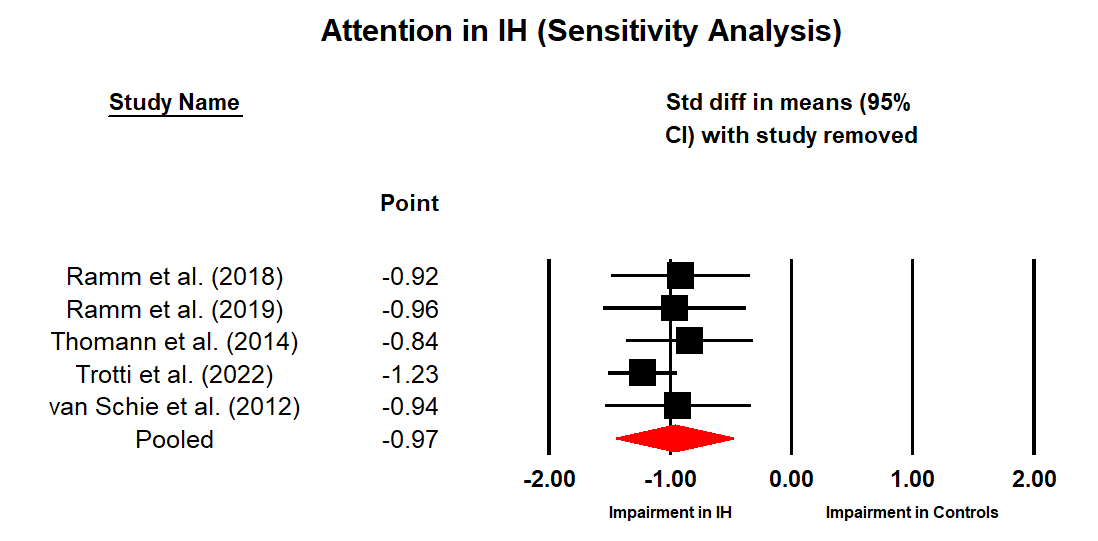

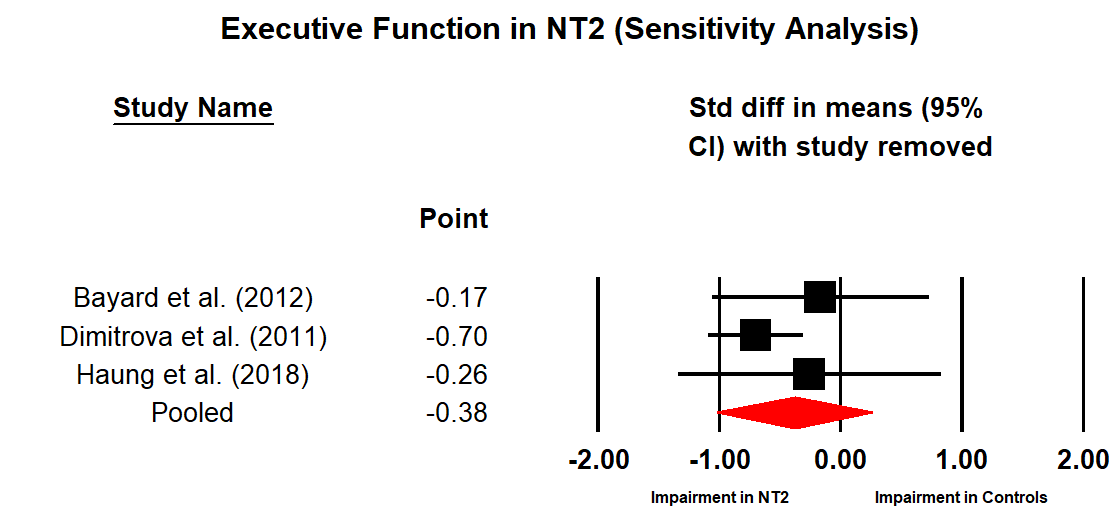


**c)**

**b)**

### **Supplementary Figure S2.** Sensitivity analysis for (a) attention and (b) executive function domains in narcolepsy type 2 (NT2) and for (c) the attention domain in idiopathic hypersomnia (IH). The sizes of the squares represent study weighting due to sample size. CI, confidence interval.

**
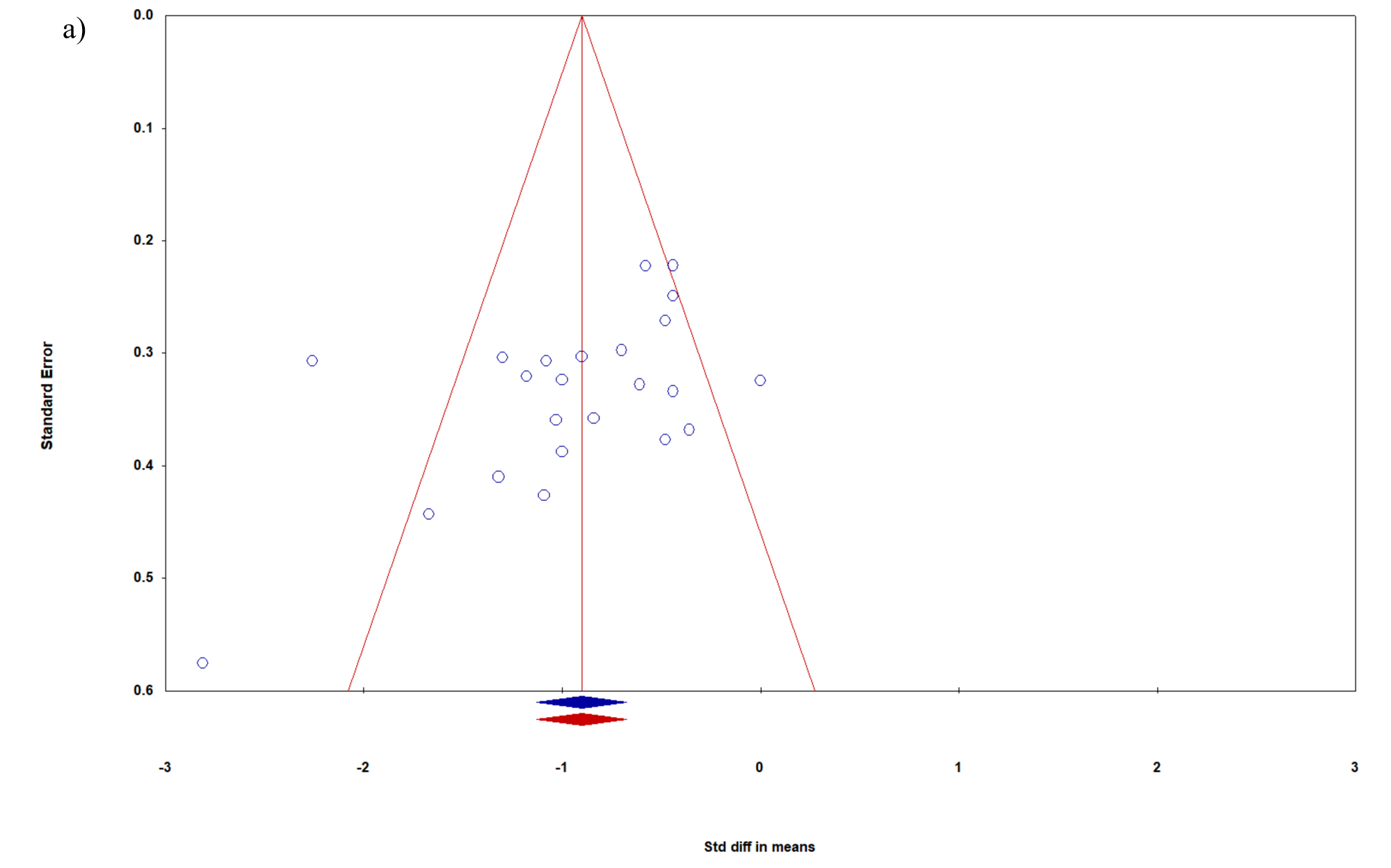
**

**a)**

**
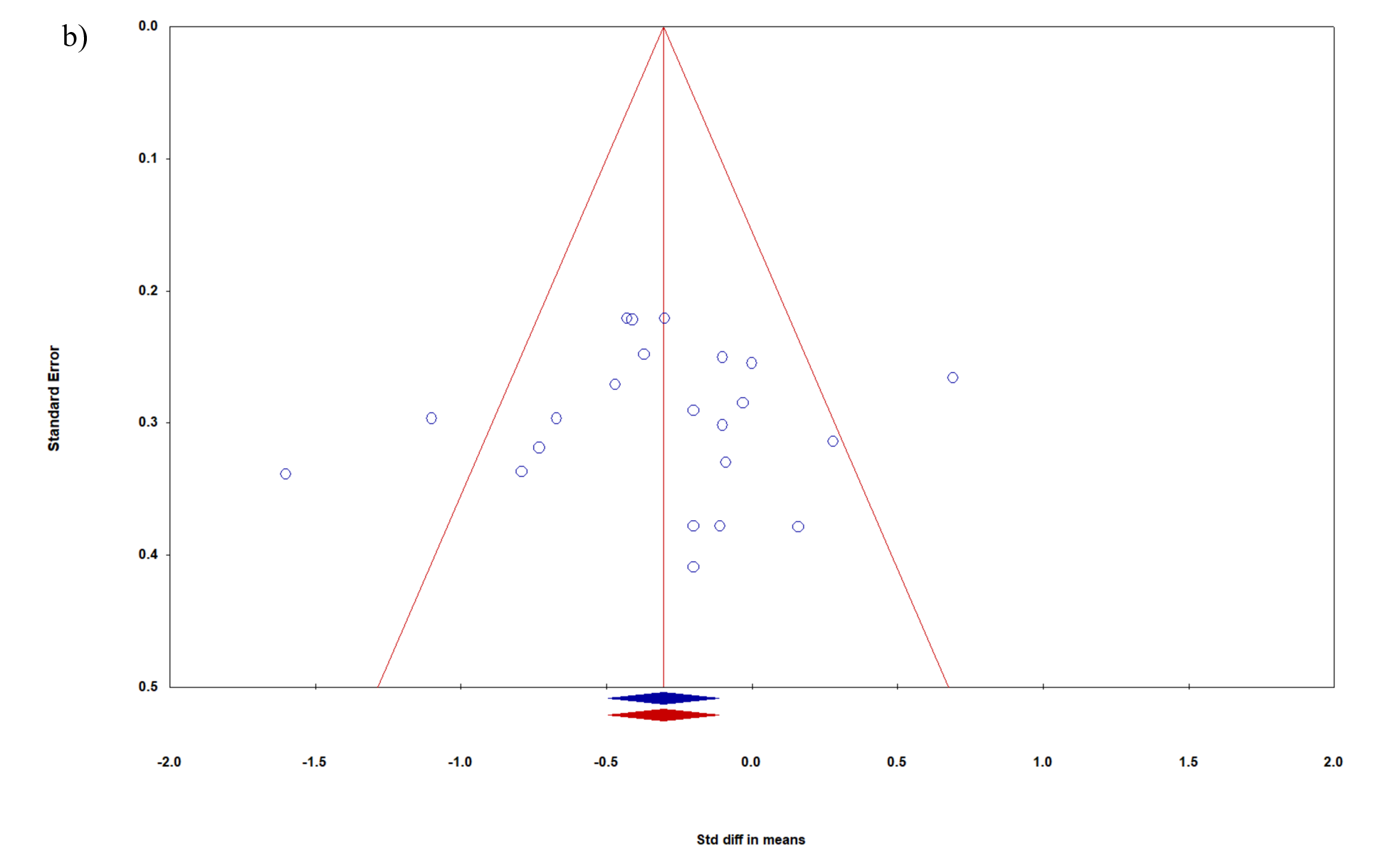
**

**b)**

**
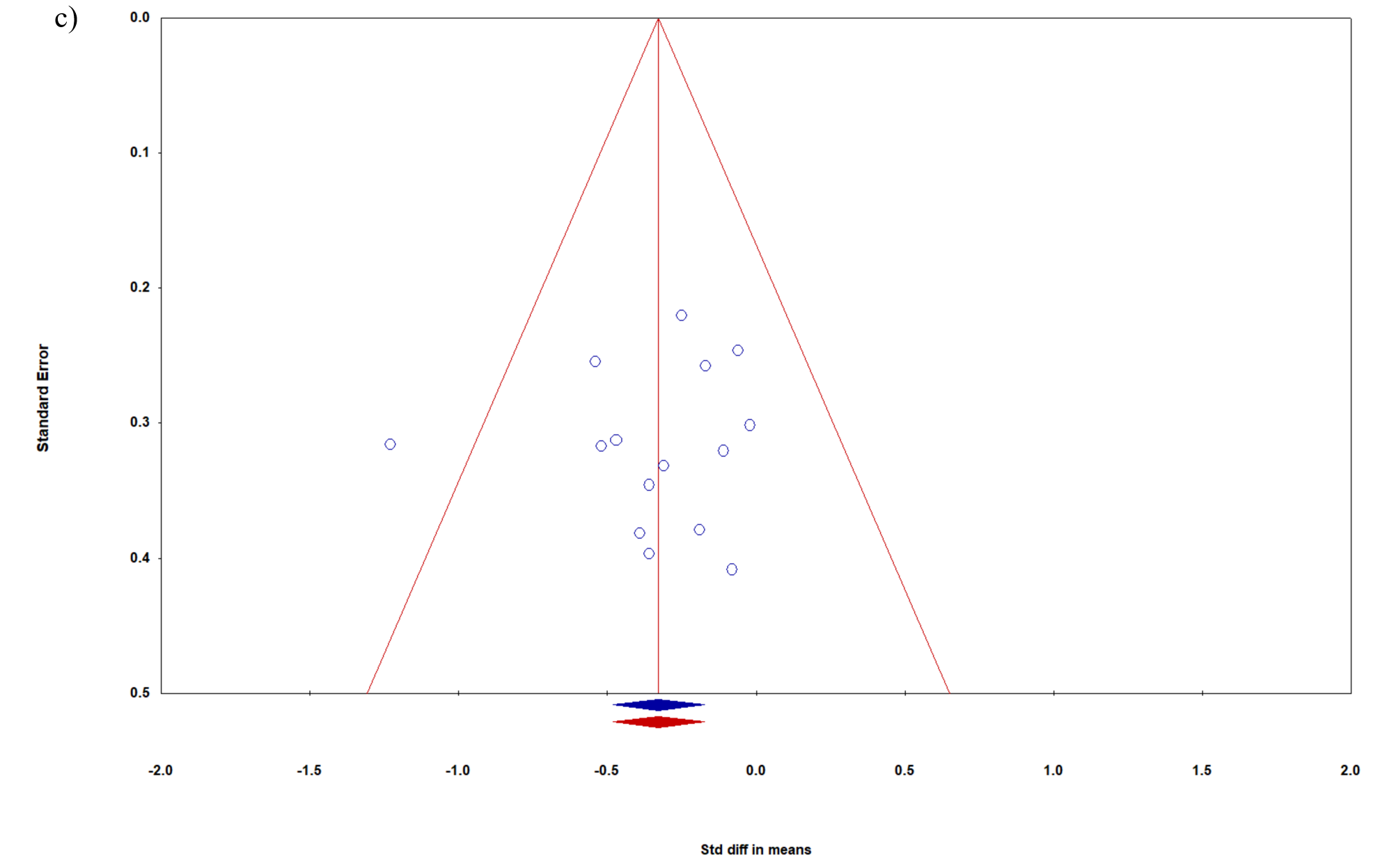
**

**c)**

### **Supplementary Figure S3.** Funnel plots of standard error by standard deviation in means for narcolepsy type 1 for (a) attention, (b) executive function, and (c) learning and memory.

**
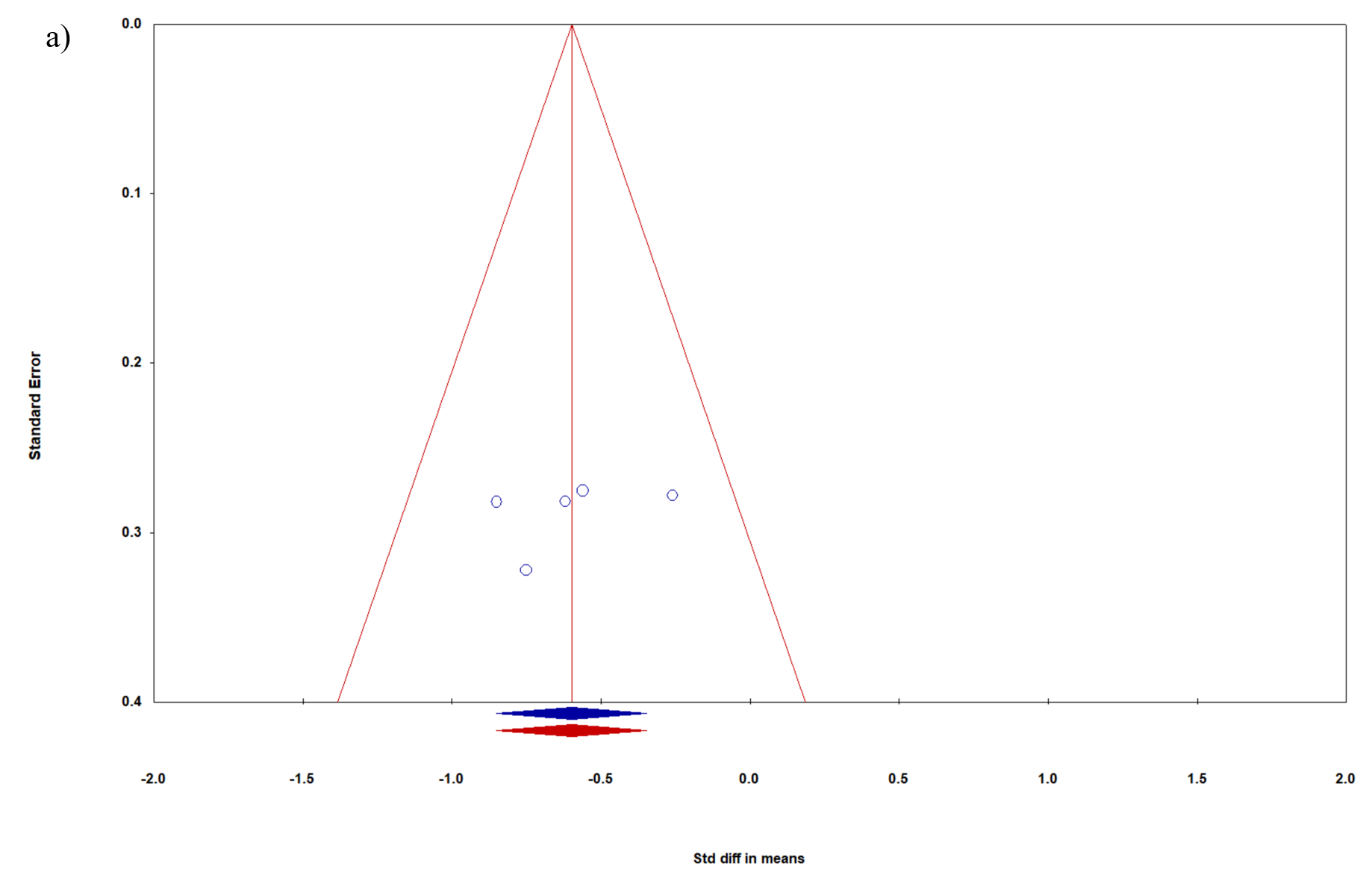
**

**a)**

**b)**

**
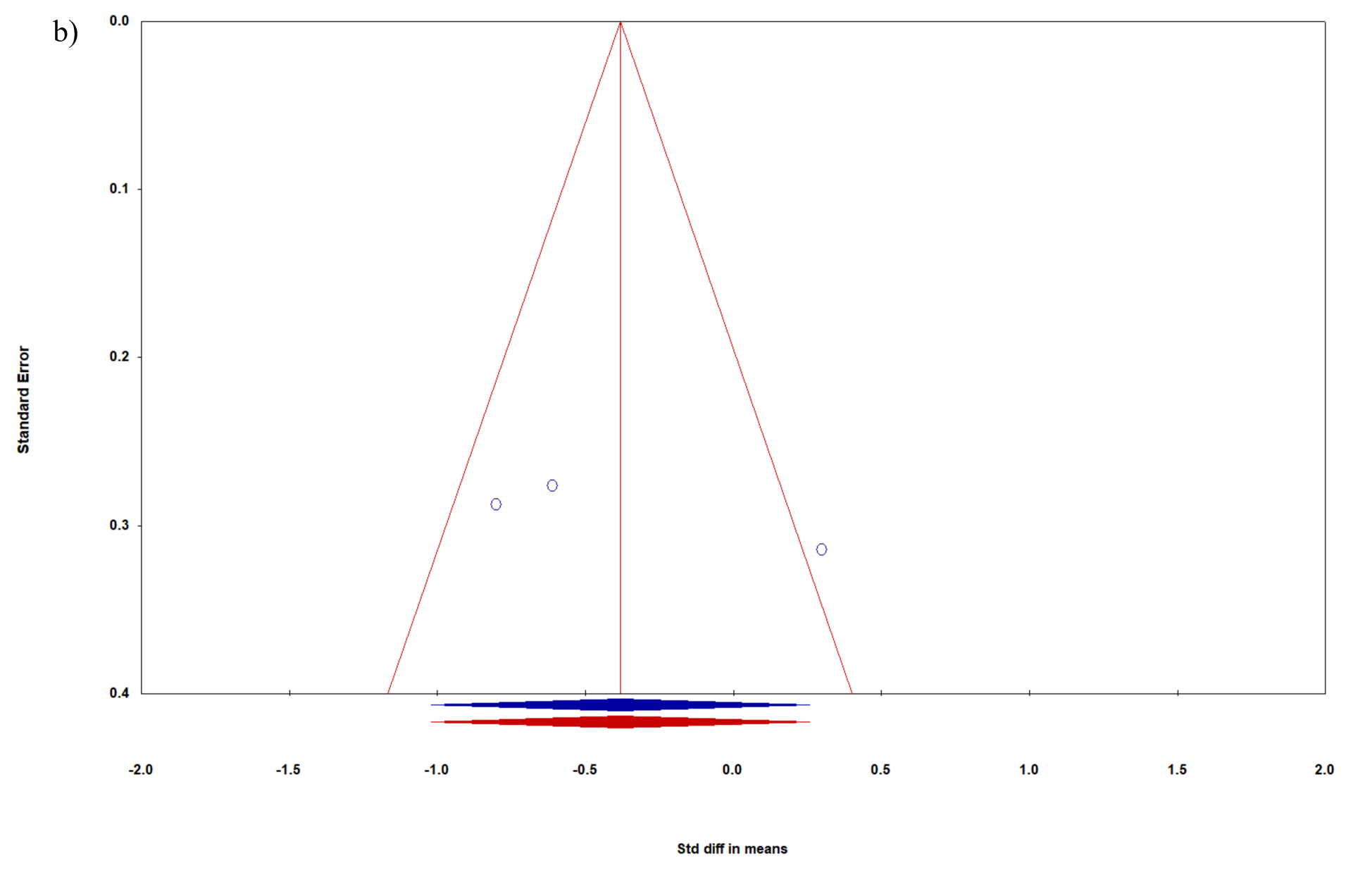
**

**c)**

**
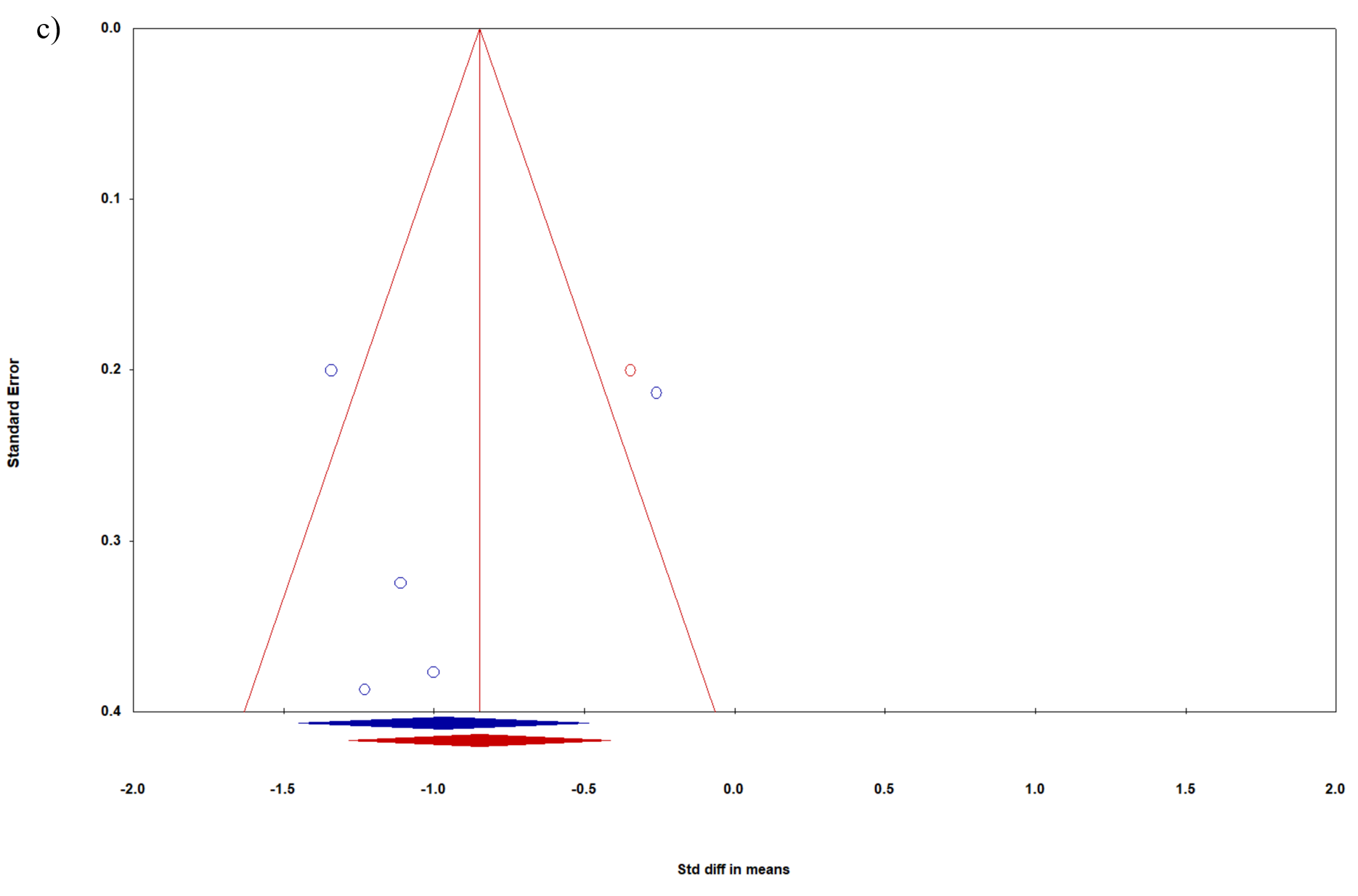
**

### **Supplementary Figure S4.** Funnel plots of standard error by standard deviation in means for narcolepsy type 2 (NT2) and idiopathic hypersomnia (IH) for (a) attention in NT2, (b) executive function in NT2, and (c) attention in IH.

## Post hoc analyses of the influence of study characteristics on pooled estimates of attention and executive function.

### **Supplementary Table S2.** Meta-regression examining the influence of moderator variables on attention in narcolepsy type 1

| **Covariate** | **No. of studies (*k*)** | **ß Coefficient (95% CI)** | **t-value df = 13** | ***p*-value** | ***R^2^*** |
| --- | --- | --- | --- | --- | --- |
| Medication use? (No) | 23 | –0.296 (–0.970 to 0.378) | –0.67 | 0.36 | 0 |
| Mean age | 23 | –0.018 (–0.056 to 0.022) | –0.94 | 0.36 | 0 |
| Continent (Europe) | 23 | –0.477 (–1.481 to 0.464) | –1.09 | 0.29 (0.43) | 0 |
| Continent (North America) | 23 | –0.617 (–2.459 to 1.225) | –0.72 | 0.482 (0.43) | 0 |
| Continent (South America) | 23 | 0.309 (–1.584 to 2.203) | 0.35 | 0.73 (0.43) | 0 |
| Male, % | 23 | –0.008 (–0.036 to 0.020) | –0.60 | 0.56 | 0 |
| No. of cognitive measures | 23 | 0.026 (–0.065 to 0.118) | 0.62 | 0.54 | 0 |
| Quality of control group | 23 | 0.017 (–0.120 to 0.155) | 0.27 | 0.79 | 0 |

Parentheses in the p-value column indicate the p-value for linked covariates. Categorical variables use a reference variable as recommended by CMA. R^2^ represents the total variance explained by the model when prior covariates are held constant.

CI, confidence interval; df, degrees of freedom.

### **Supplementary Table S3.** Meta-regression examining the influence of moderator variables on executive function in idiopathic hypersomnia

| **Covariate** | **No. of studies (*k*)** | **ß Coefficient (95% CI)** | **t-value**  **df = 14** | ***p*-value** | ***R^2^*** |
| --- | --- | --- | --- | --- | --- |
| Medication use? (No) | 22 | –0.771 (–1.610 to 0.068) | –1.97 | 0.07 | 0.05 |
| Mean age | 22 | –0.014 (–0.061 to 0.032) | –0.66 | 0.52 | 0.11 |
| Continent (Europe) | 22 | –0.296 (–1.077 to 0.485) | –0.81 | 0.43 (0.62) | 0.08 |
| Continent (South America) | 22 | –0.532 (–1.857 to 0.792) | –0.86 | 0.403 (0.62) | 0.09 |
| Male, % | 22 | 0.016 (–0.013 to 0.045) | 1.18 | 0.26 | 0.00 |
| No. of cognitive measures | 22 | –0.114 (–0.241 to 0.013) | –1.92 | 0.08 | 0.17 |
| Quality of control group | 22 | –0.059 (–0.192 to 0.074) | –0.95 | 0.359 | 0.18 |

Parentheses in the p-value column indicate the p-value for linked covariates. Categorical variables use a reference variable as recommended by CMA. R^2^ represents the total variance explained by the model when prior covariates are held constant.

CI, confidence interval; df, degrees of freedom.

# Study 2: Post hoc meta-analyses of cognitive impairment in NT1, NT2, and IH


## Classification of cognitive tests according to attentional functions.

The cognitive functions identified were then reorganized into their attentional subdomain of either sensory selective attention, controlled attention, focused attention, or sustained attention in accordance with the neuropsychological framework (Table S4).

### **Supplementary Table S4.** Summary of attentional tests used in studies of narcolepsy type 1, narcolepsy type 2, and idiopathic hypersomnia, classified according to the attentional subdomain assessed

| **Attentional subdomain** | **Neuropsychological test** |
| --- | --- |
| Sensory selective attention | Visual Scanning Task [7]  Visual Tracking Test [9]  Attention Network Test (Orienting) [6]  Tonic Alertness [2] |
| Controlled attention | Complex and Multiple Response Time Tasks [5]  Attention Network Test (Executive) [6]  Digit Symbol Test [23]  d2 Test [1,18]  Bourdon Vos Task [17] |
| Focused attention | Simple Reaction Time Tasks [1-4]  Forward Digit Span [1,5,19,20]  Divided Attention Task [3]  Selective Attention Task [3,8]  Visual and Acoustic Parallel Tasks [1]  Single and Dual Visual and Auditory Tasks [7]  Psychomotor Activity [35]  Visual Tracking Test [9] |
| Sustained attention/vigilance | Psychomotor Vigilance Task [10-12,31]  Vigilance Task [2]  Continuous Performance Test [37,38]  Sustained Attention Task [3]  Sustained Attention to Response Task [12,14-17]  Attention Network Task (Alerting) [6]  Alertness Task [7] |

## Contribution of data from individual tests to post hoc pooled estimates of impairment in each attention function determined from meta-analysis.


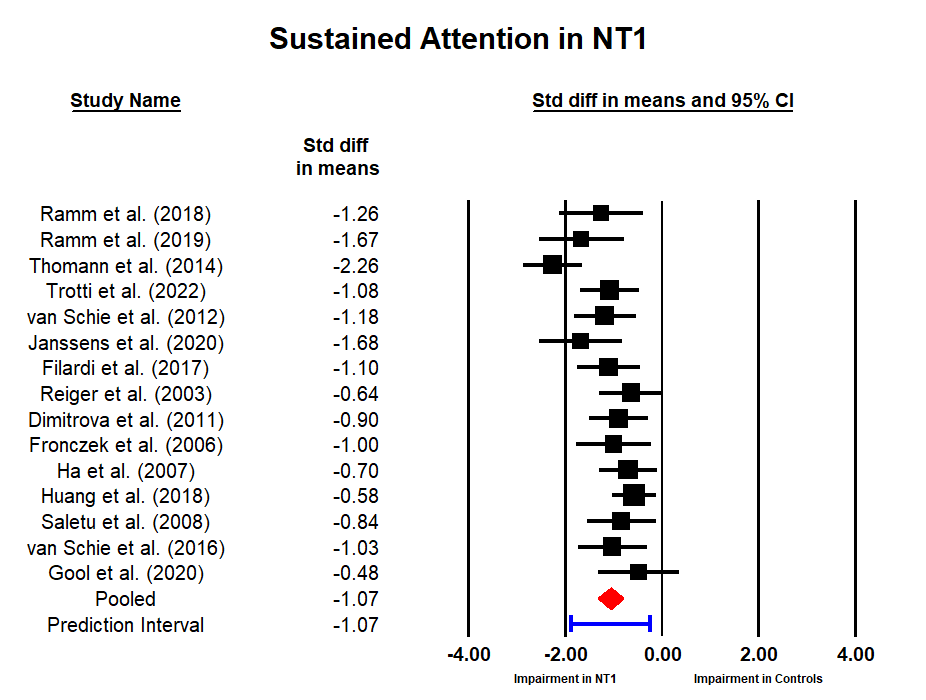
**a)**


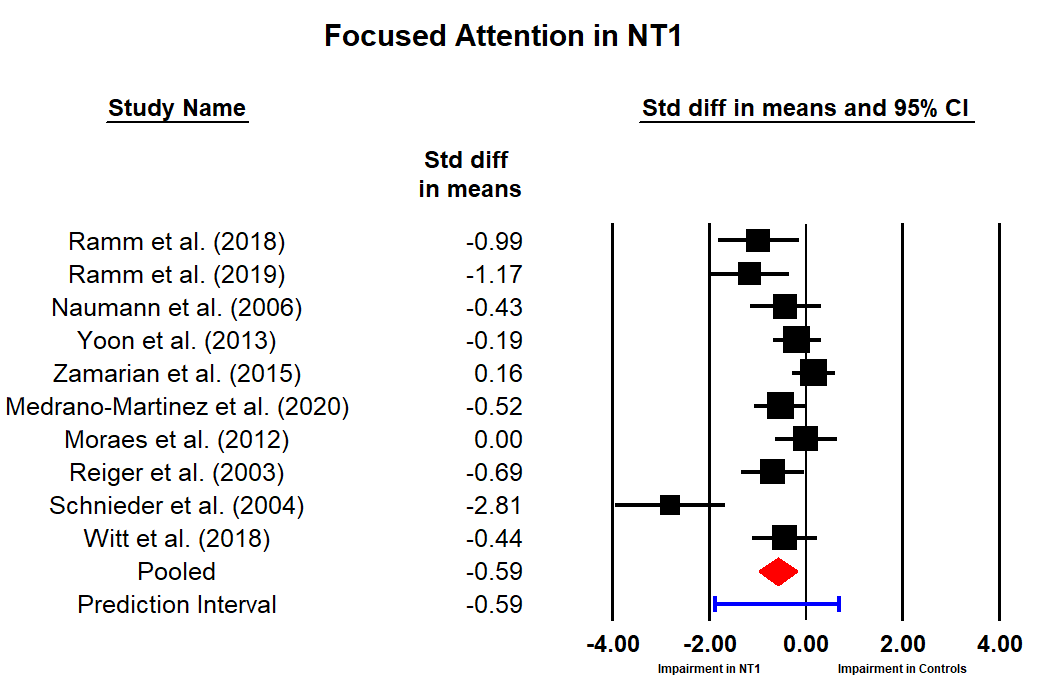


**b)**

**
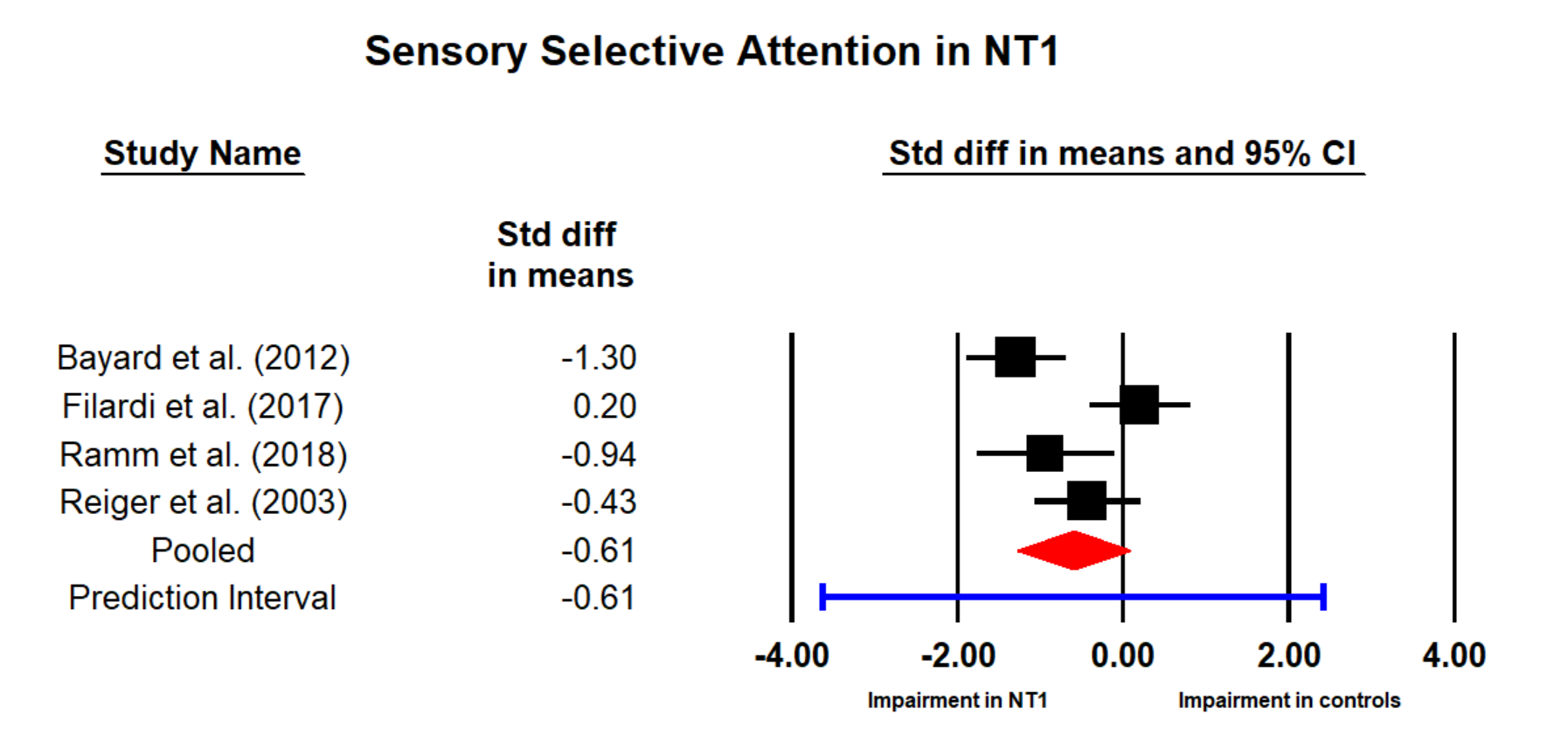
c)**


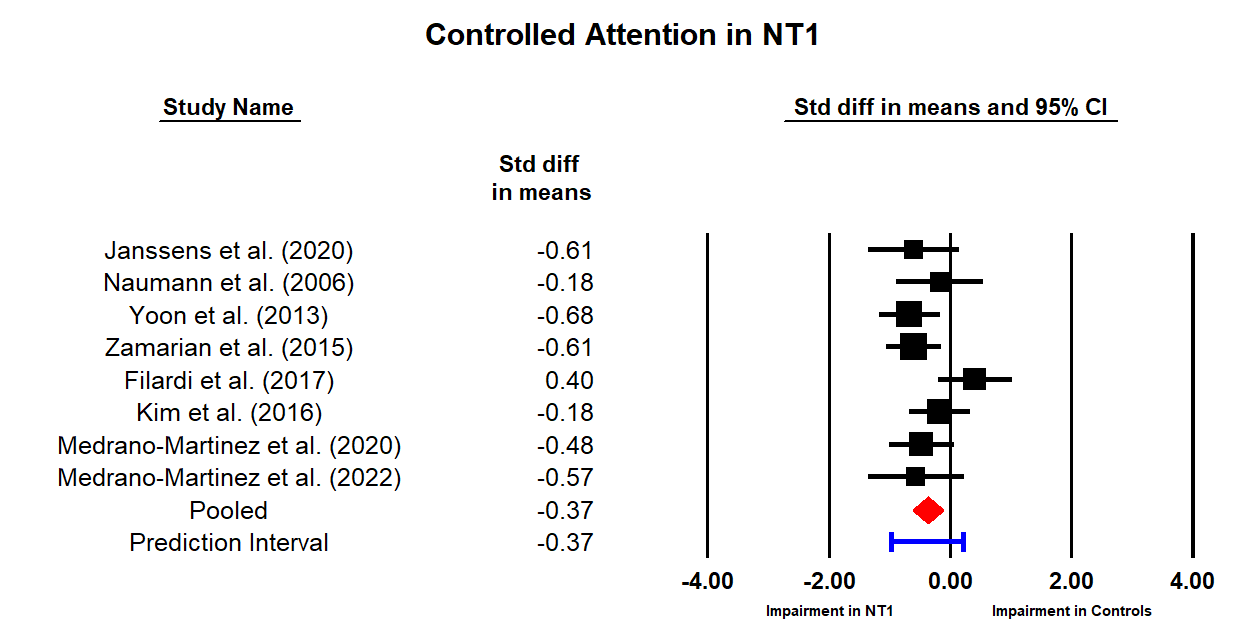


**d)**

### **Supplementary Figure S5.** Individual and pooled effect sizes of neuropsychological tests of attention subdomains in narcolepsy type 1 (NT1). The sizes of the squares represent study weighting due to sample size. CI, confidence interval.

**
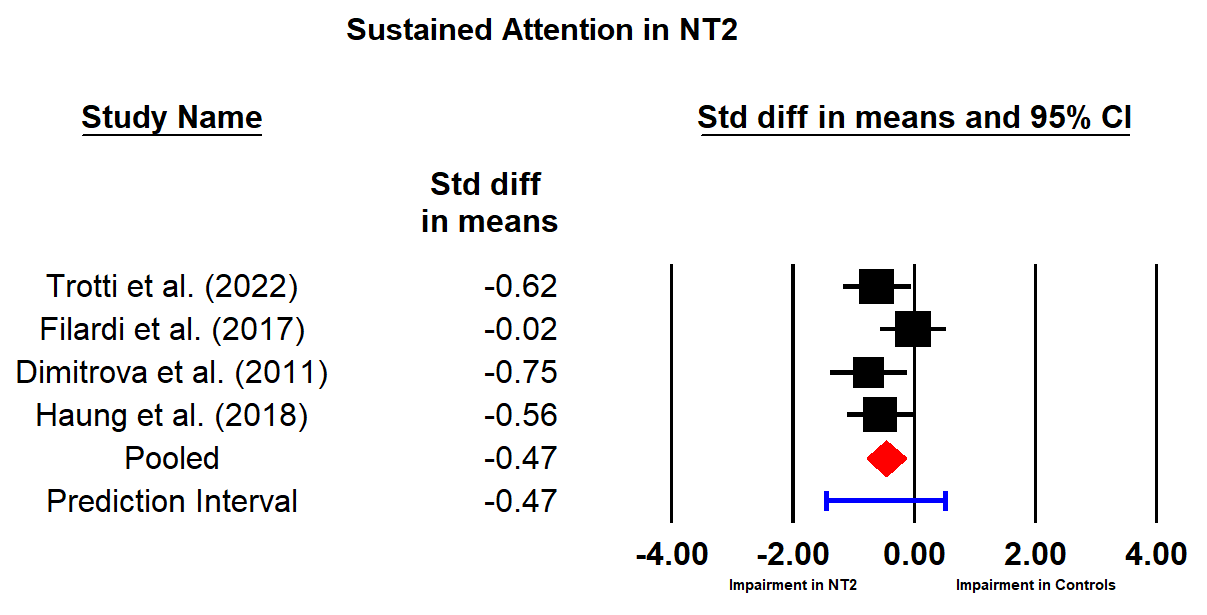
a)**


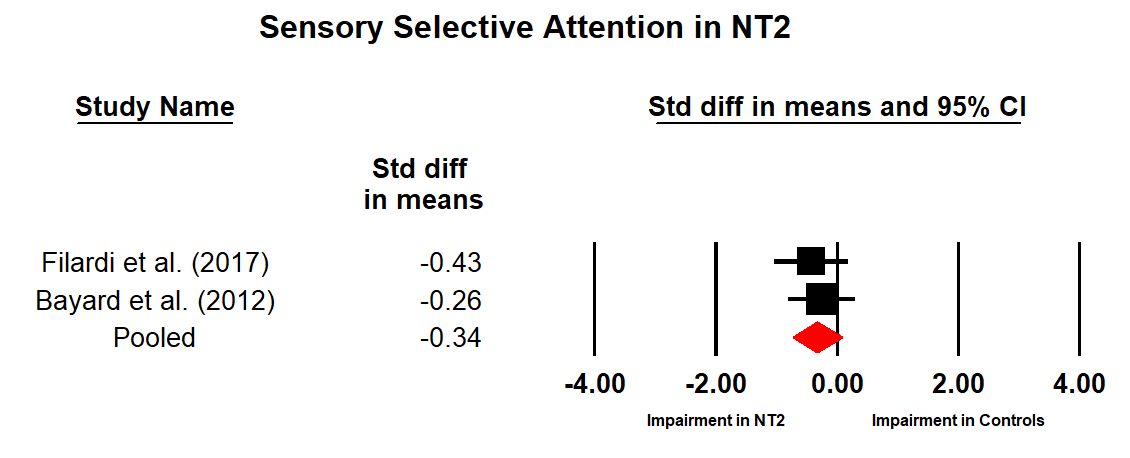
**b)**


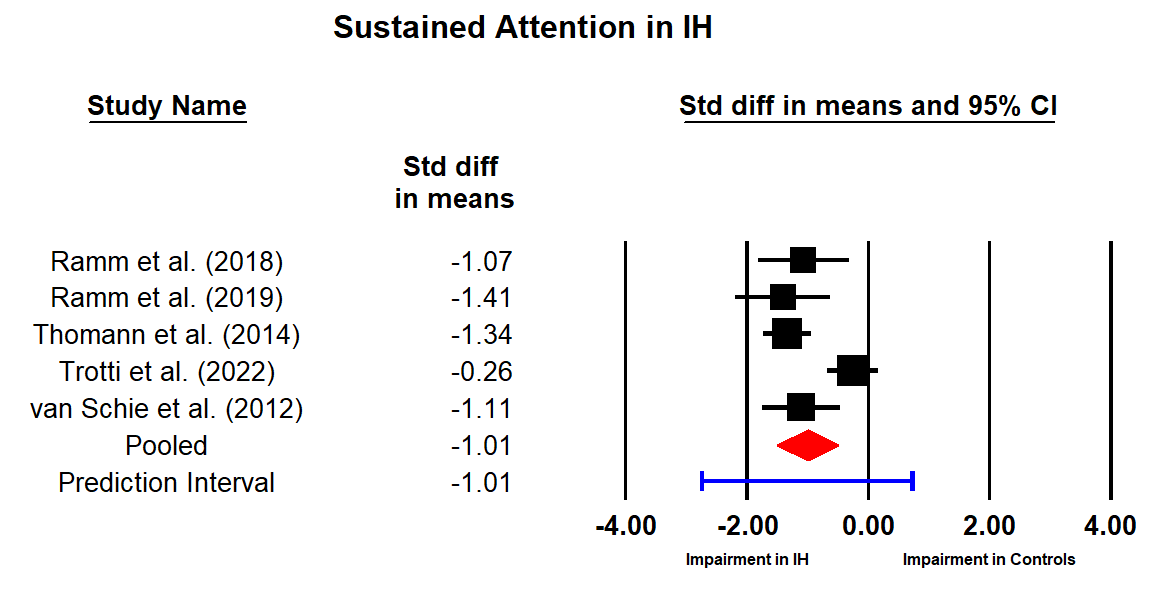
**c)**


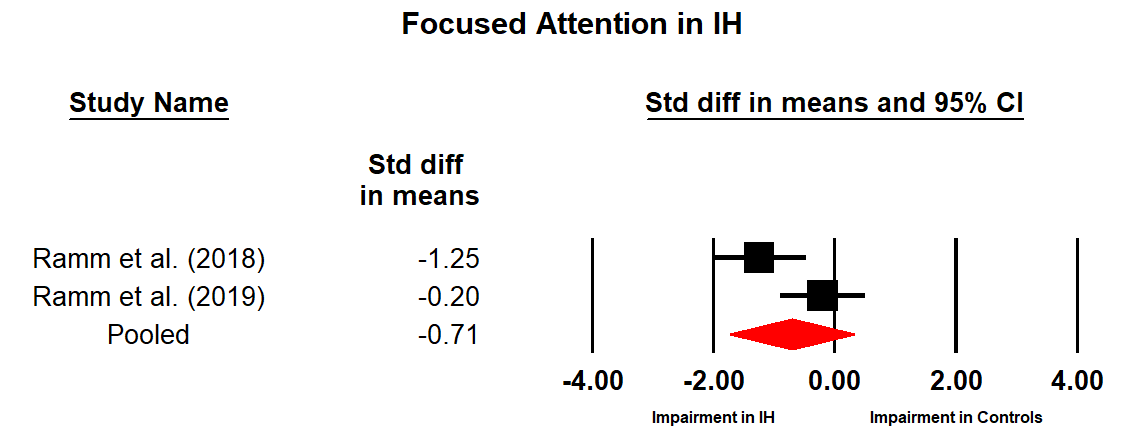
**d)**

### **Supplementary Figure S6.** Individual and pooled effect sizes of neuropsychological tests of attention subdomains in narcolepsy type 2 (NT2) and idiopathic hypersomnia (IH). The sizes of the squares represent study weighting due to sample size. CI, confidence interval.

## Post hoc analyses of sensitivity of post hoc pooled estimates of impairment in attentional functions to the influence of single studies and publication bias.


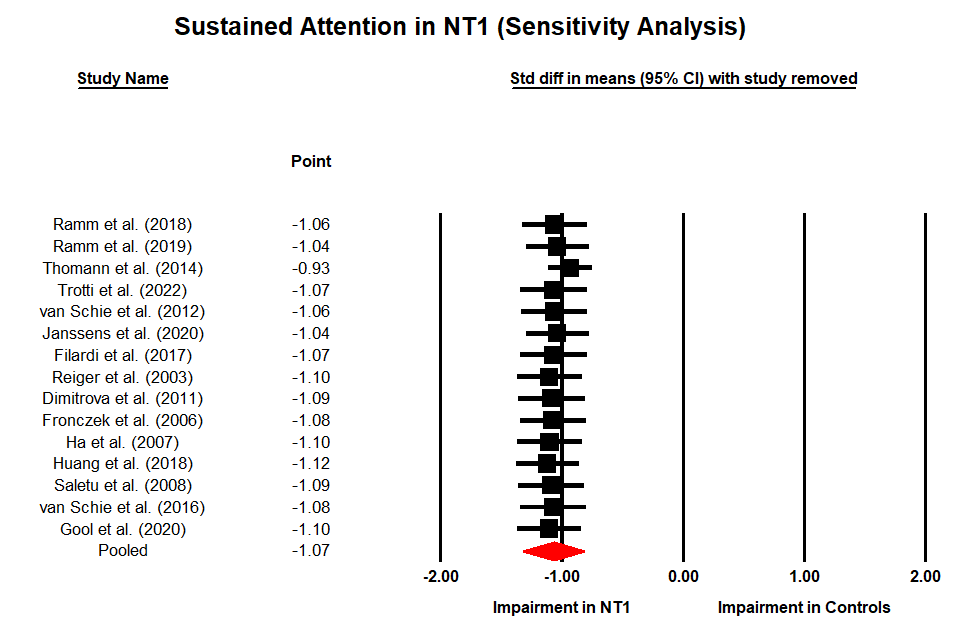


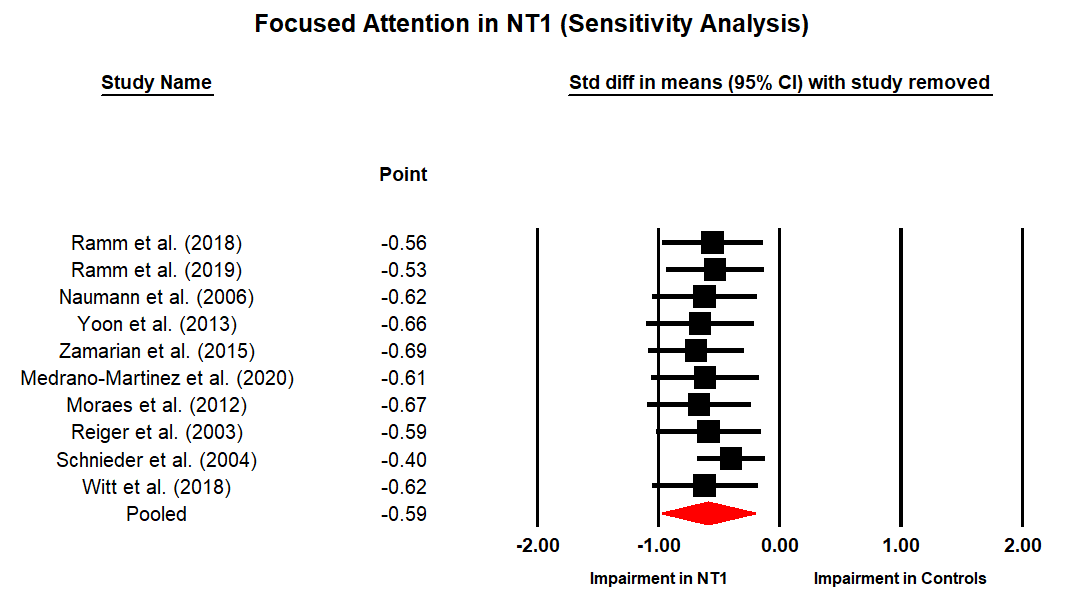


**
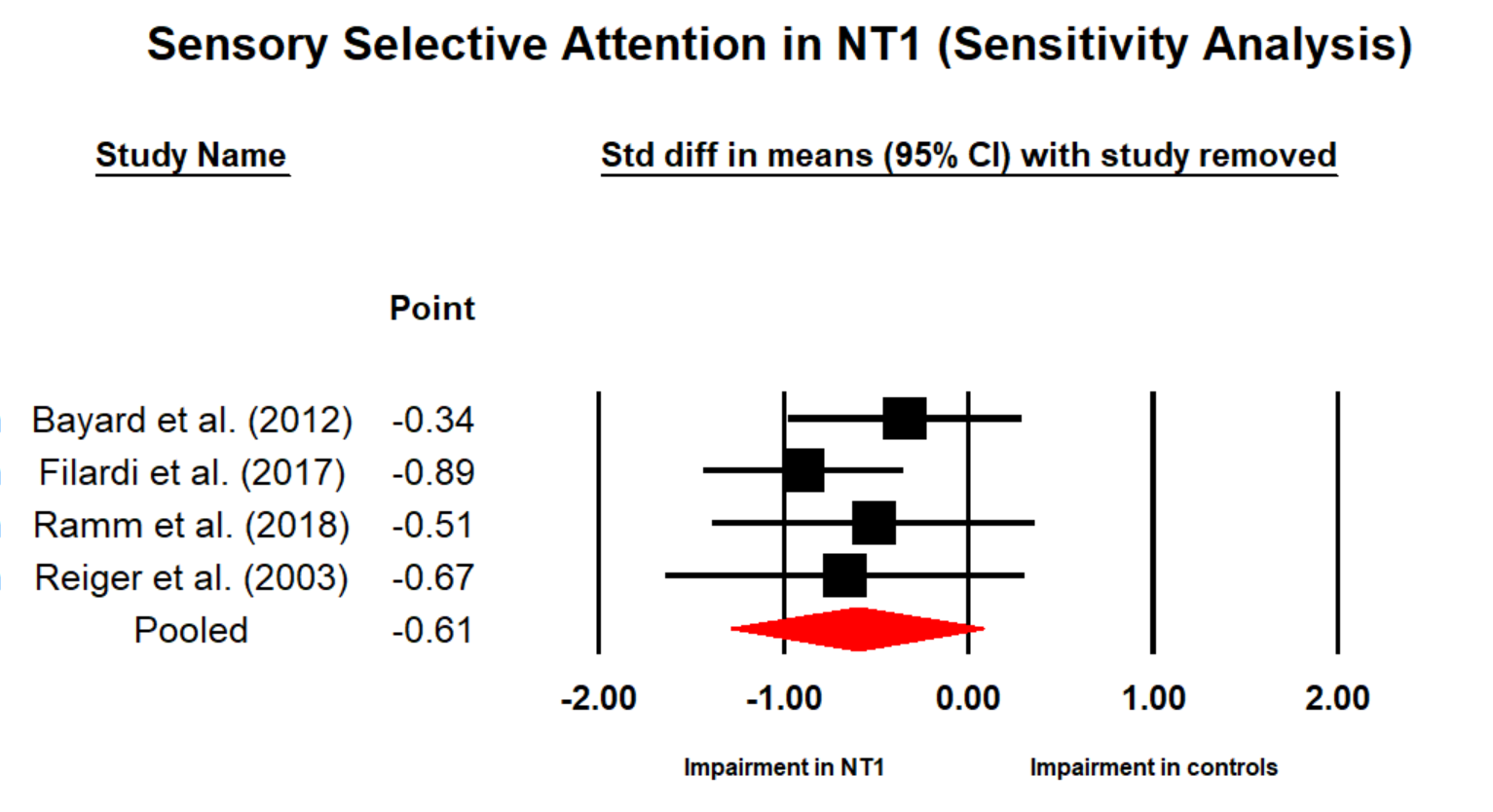
**


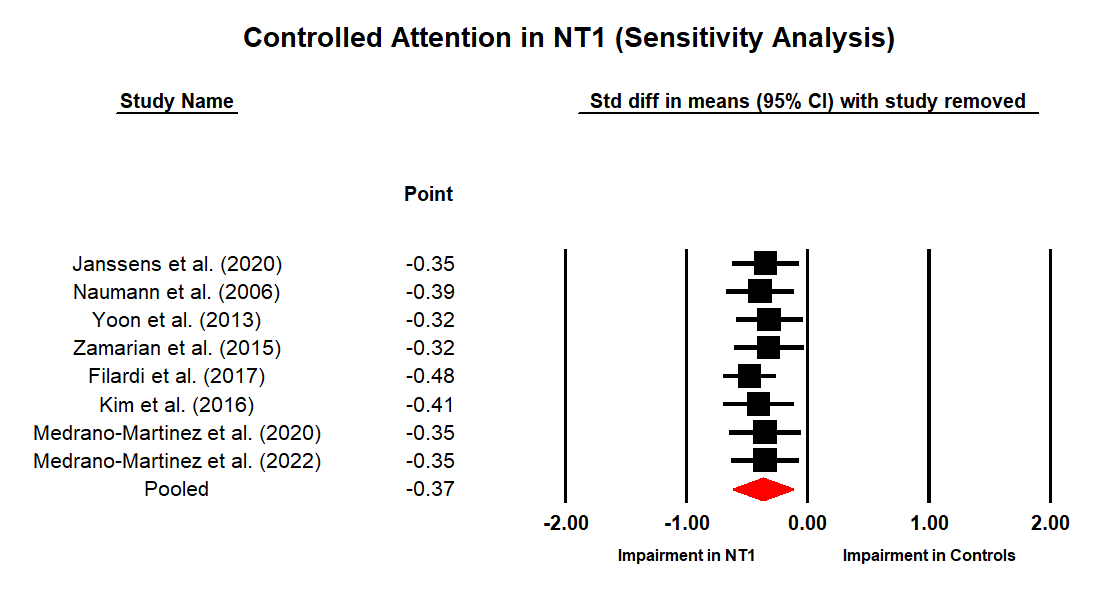


### **Supplementary Figure S7.** Sensitivity analysis of attention subdomains in narcolepsy type 1 (NT1). CI, confidence interval.


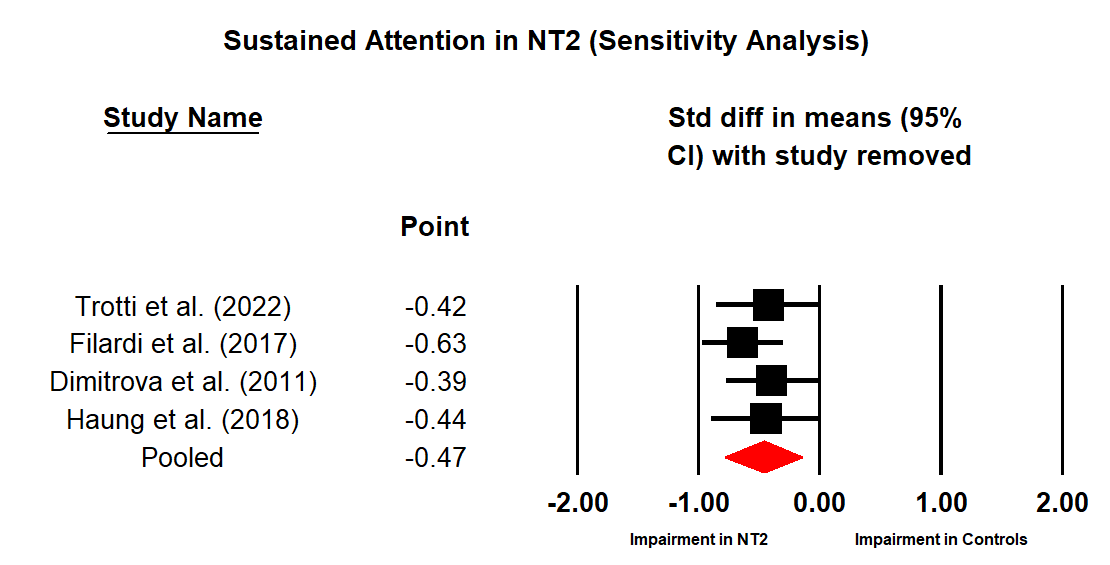


**
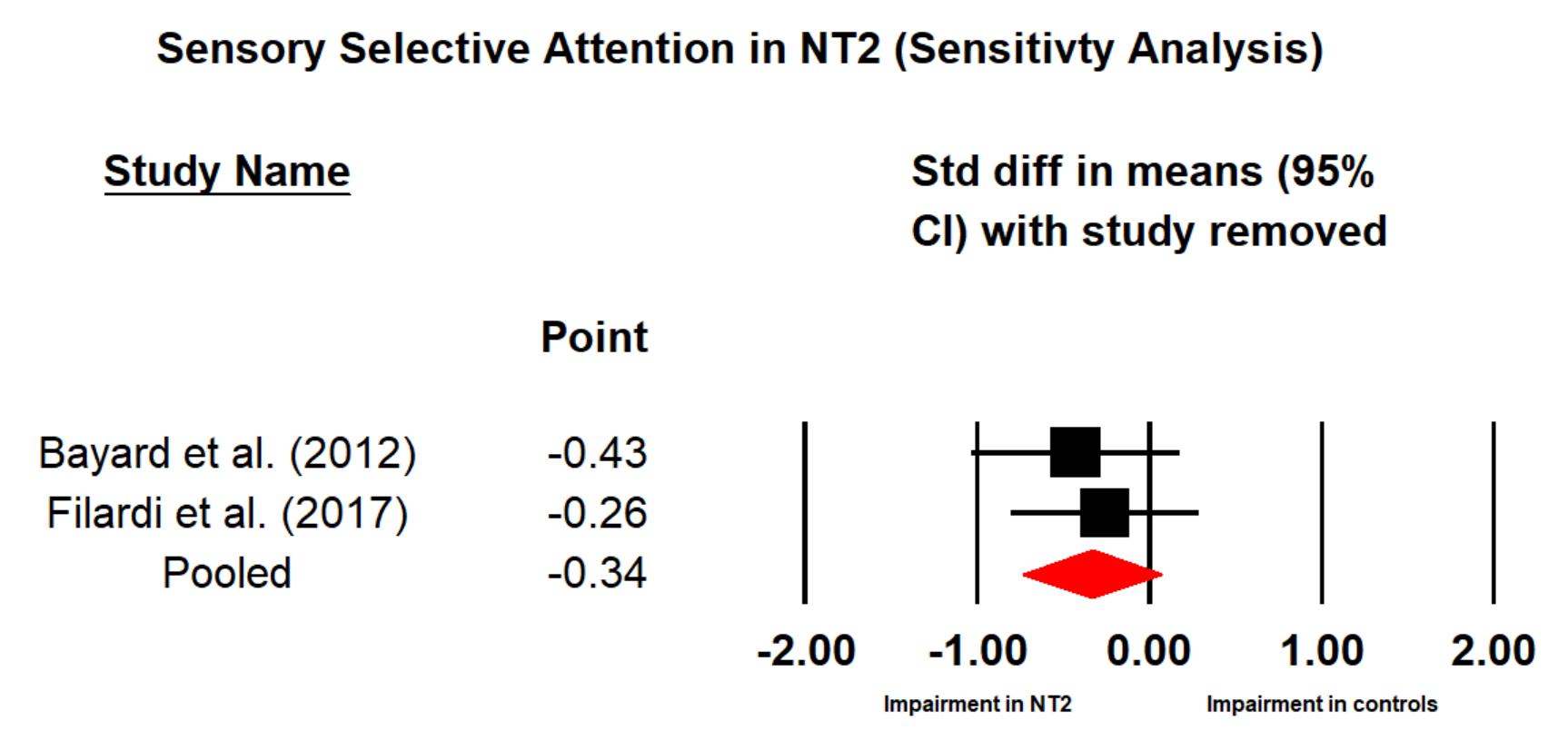
**


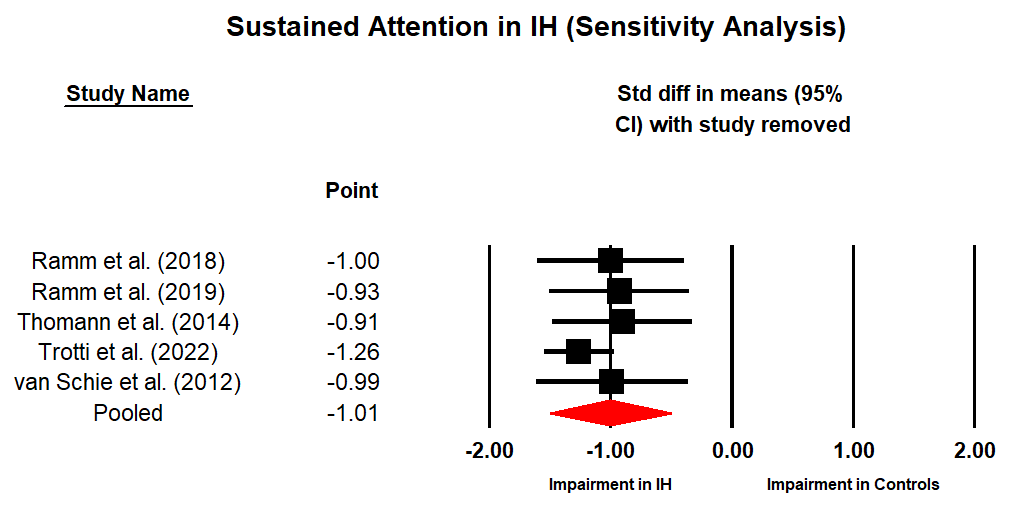


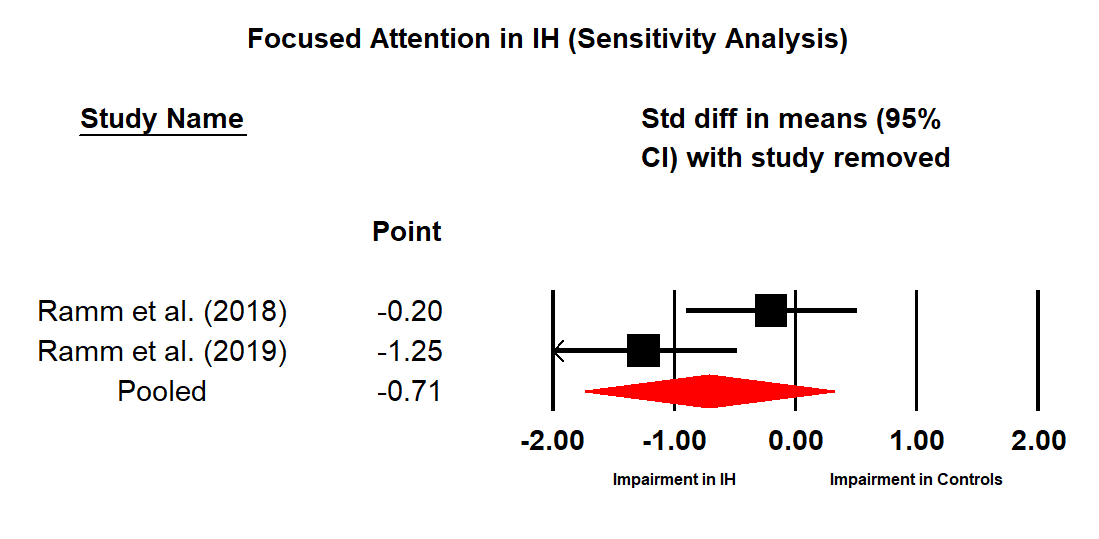


### **Supplementary Figure S8.** Sensitivity analysis of attention subdomains in narcolepsy type 2 (NT2) and idiopathic hypersomnia (IH). CI, confidence interval.


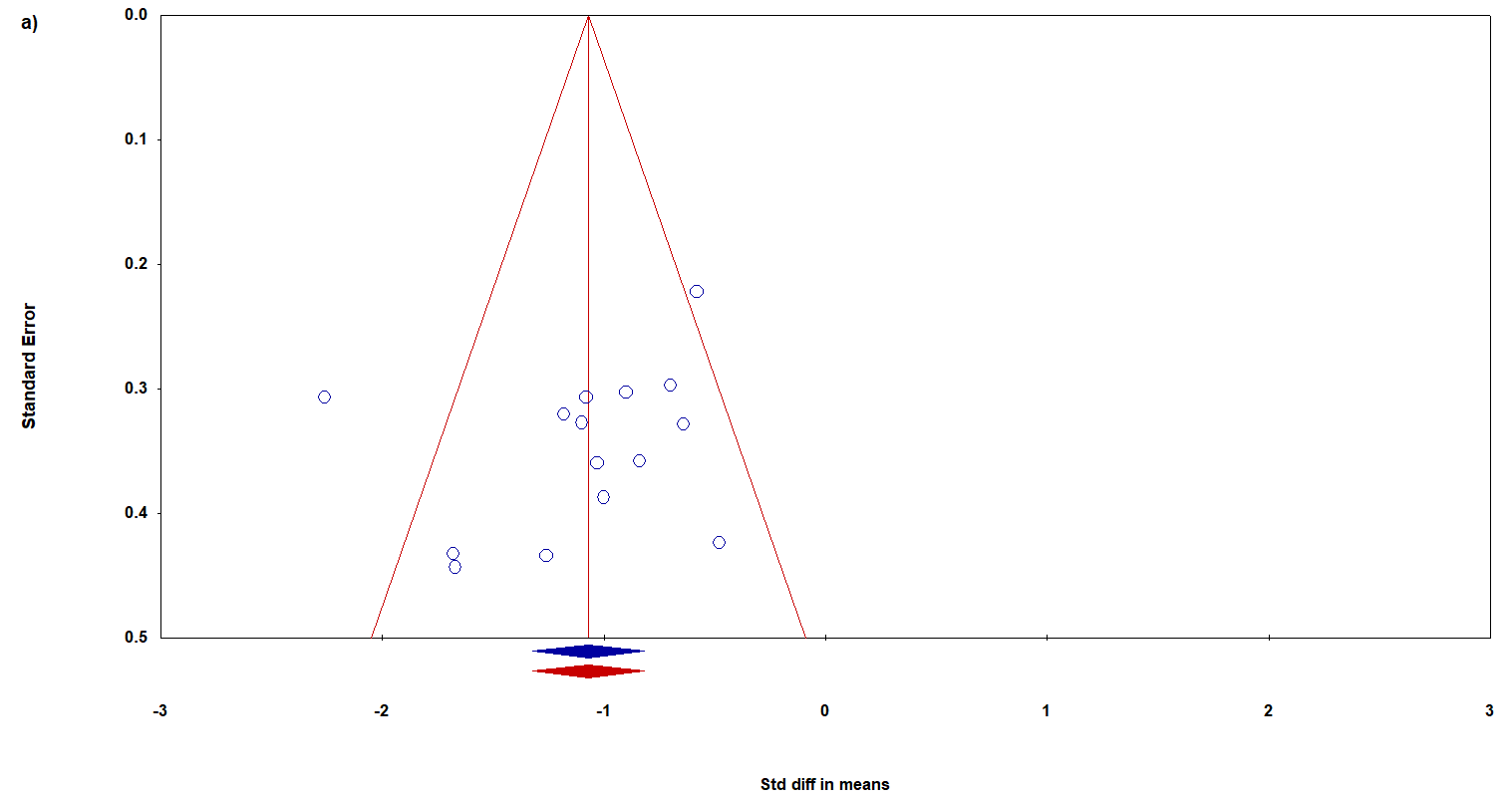


**a)**


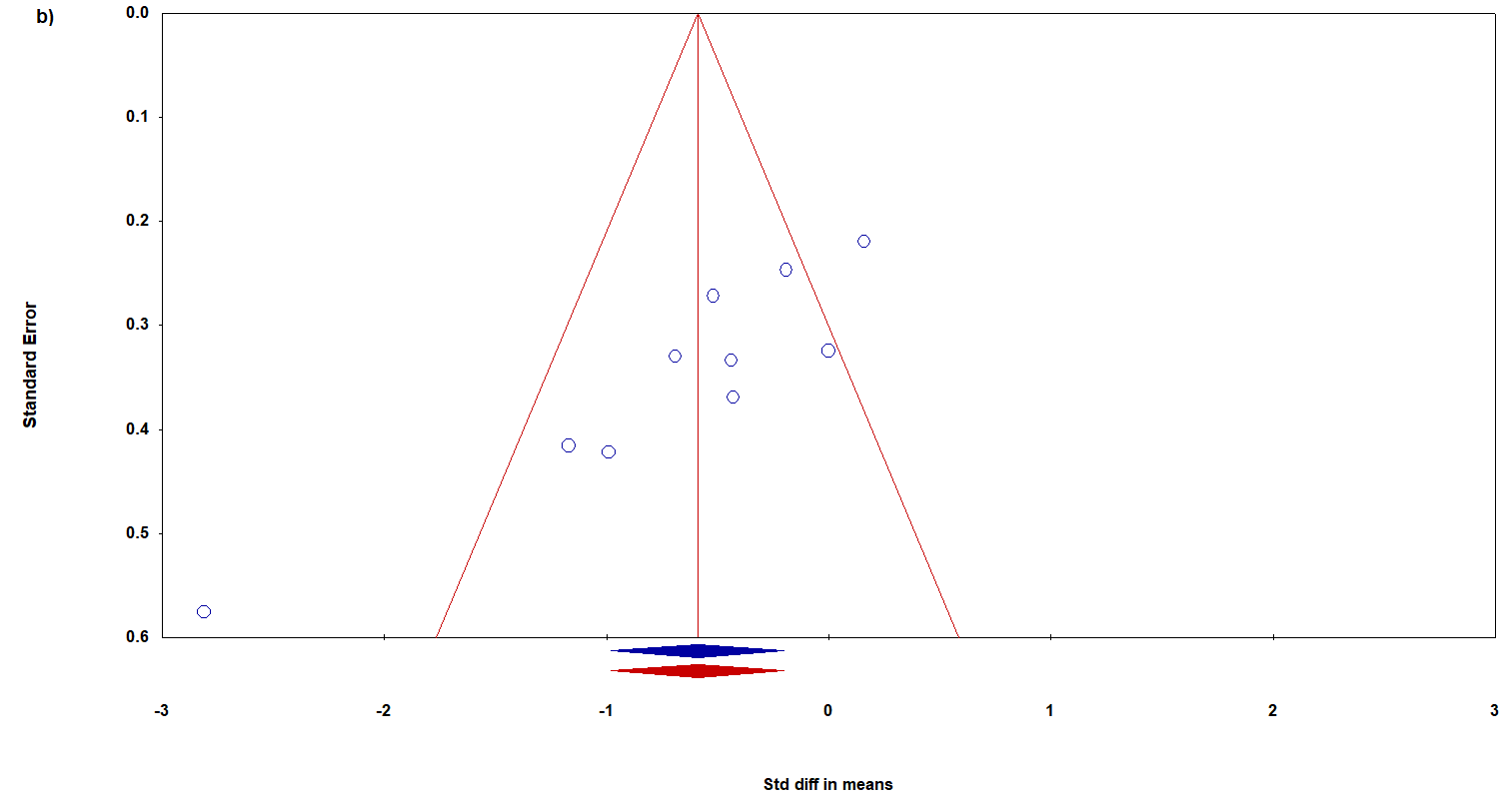


**b)**


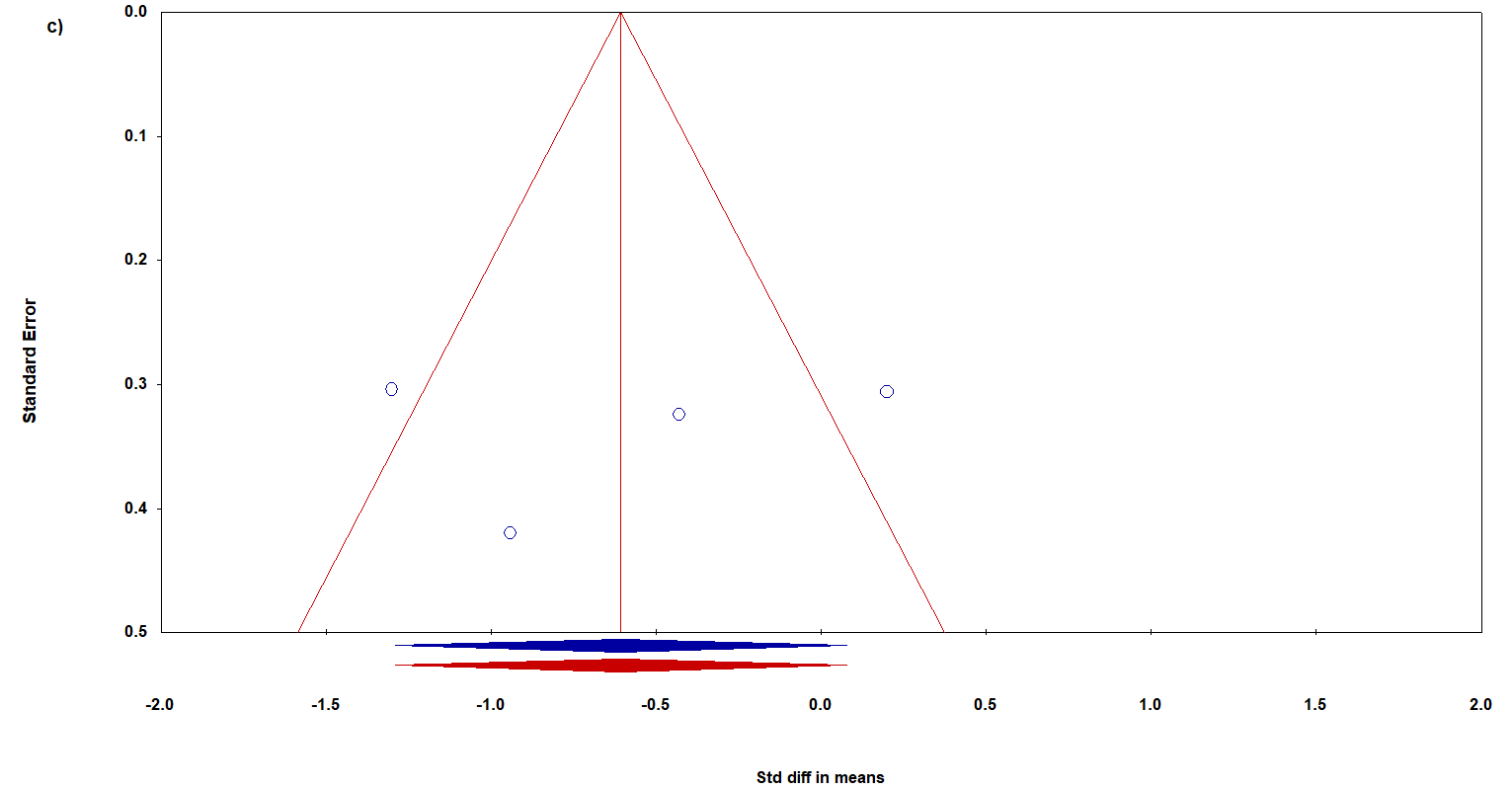


**c)**


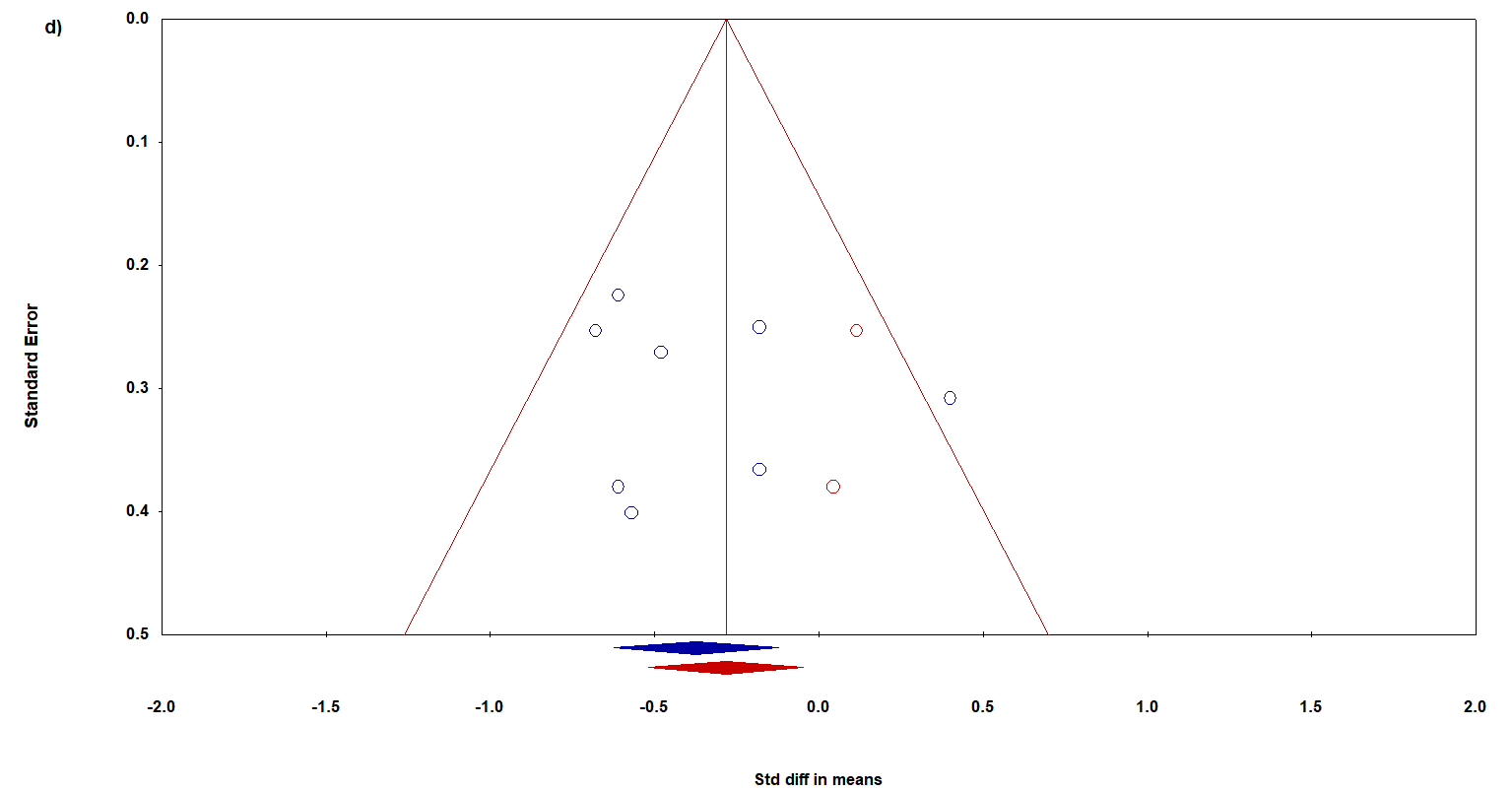


**d)**

### **Supplementary Figure S9.** Funnel plots for attentional subdomains in narcolepsy type 1: (a) sustained attention, (b) focused attention, (c) sensory selective attention, and (d) controlled attention.


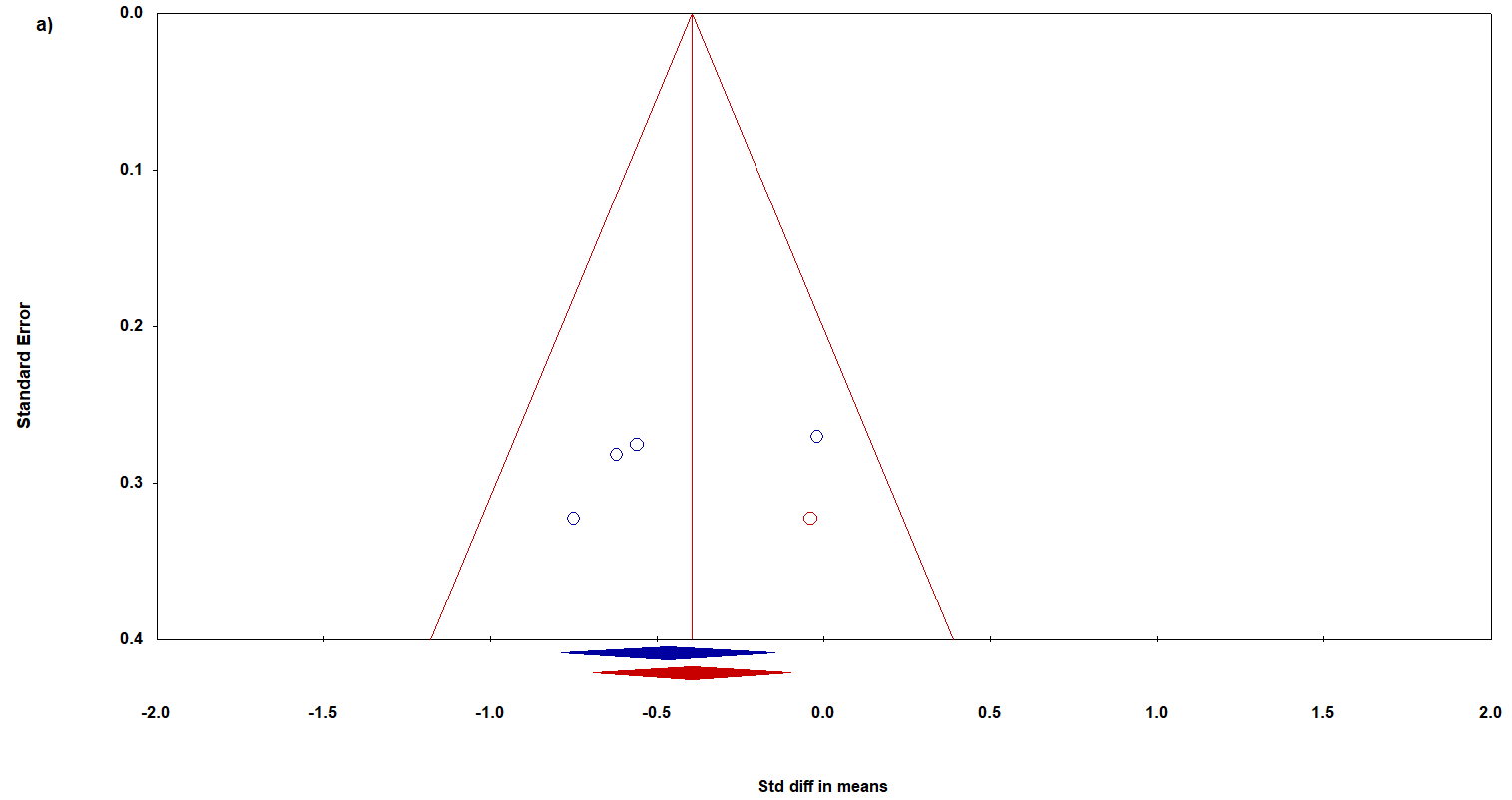


**a)**


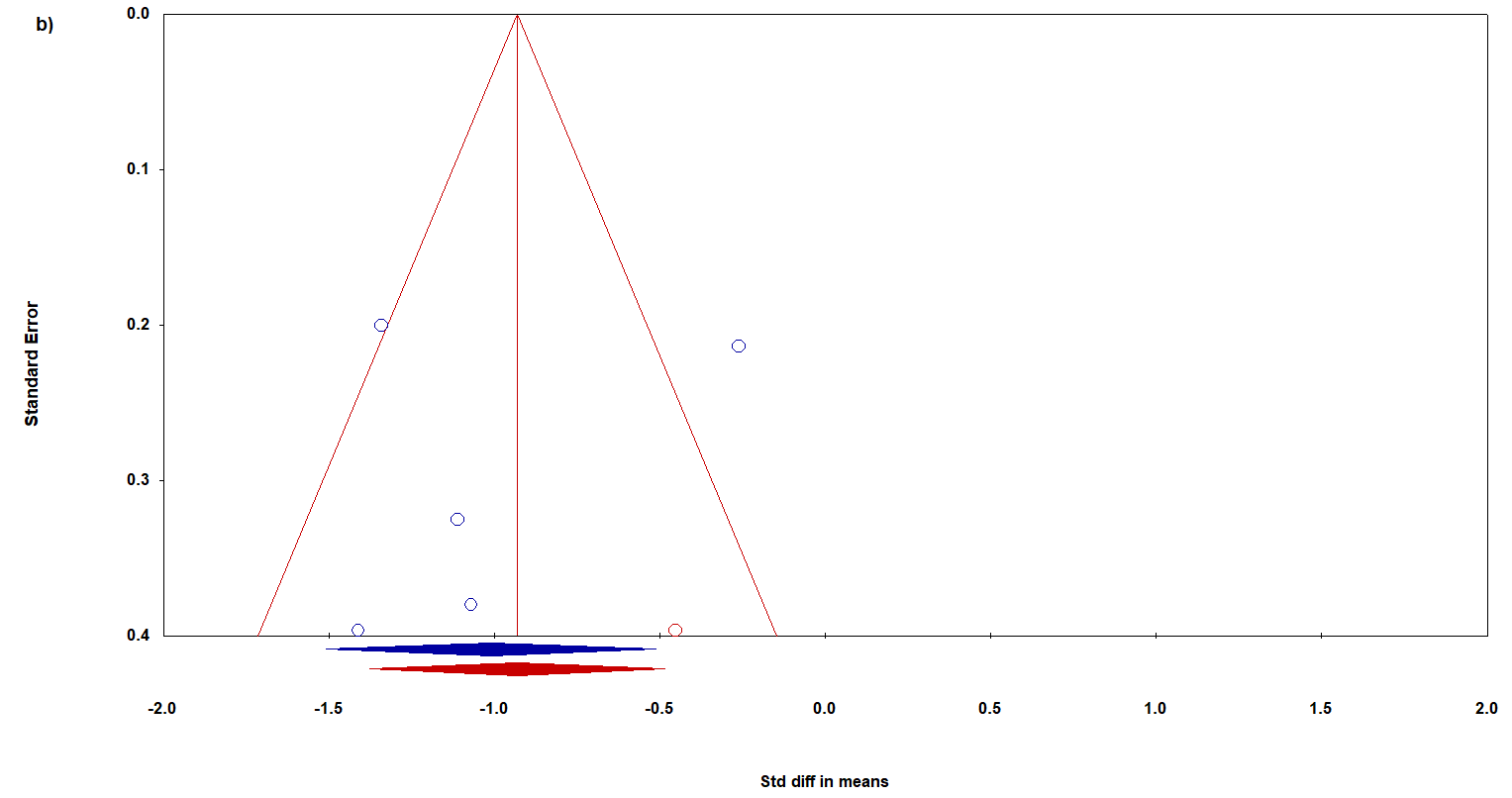


**b)**

### **Supplementary Figure S10.** Funnel plots of attentional subdomains in narcolepsy type 2 (NT2) and idiopathic hypersomnia (IH): (a) sustained attention in NT2 and (b) sustained attention in IH.

### **Supplementary Table S5.** Meta-regression examining the influence of moderator variables on focused attention in narcolepsy type 1

| **Covariate** | **No. of studies (*k*)** | **ß Coefficient (95% CI)** | **t-value df = 7** | ***p*-value** | ***R^2^*** |
| --- | --- | --- | --- | --- | --- |
| Medication use? (No) | 15 | –0.118 (–1.294 to 1.057) | –0.24 | 0.82 | 0 |
| Mean age | 15 | 0.022 (–0.041 to 0.084) | 0.81 | 0.44 | 0 |
| Continent (Europe) | 15 | –0.489 (–1.856 to 0.878) | –0.85 | 0.43 (0.67) | 0 |
| Continent (North America) | 15 | –0.372 (–2.708 to 1.964) | –0.38 | 0.72 (0.67) | 0 |
| Male, % | 15 | 0.005 (–0.033 to 0.041) | 0.29 | 0.78 | 0 |
| No. of cognitive measures | 15 | 0.016 (–1.04 to 0.137) | 0.32 | 0.76 | 0 |
| Quality of control group | 15 | 0.055 (–0.116 to 0.225) | 0.75 | 0.47 | 0 |

Parentheses in the p-value column indicate the p-value for linked covariates. Categorical variables use a reference variable as recommended by CMA. R^2^ represents the total variance explained by the model when prior covariates are held constant.

CI, confidence interval; df, degrees of freedom.

### **Supplementary Table S6.** Meta-regression examining the influence of moderator variables on sustained attention in idiopathic hypersomnia

| **Covariate** | **No. of studies (*k*)** | **ß Coefficient (95% CI)** | **t-value df = 2** | ***p*-value** | ***R^2^*** |
| --- | --- | --- | --- | --- | --- |
| Medication use? (No) | 10 | –1.264 (–8.000 to 5.468) | –0.81 | 0.504 | 0 |
| Mean age | 10 | –0.062 (–0.303 to 0.180) | –1.10 | 0.387 | 0 |
| Continent (Europe) | 10 | –1.573 (–9.792 to 6.646) | –0.82 | 0.497 | 0 |
| Continent (South America) | 10 | –0.681 (–12.078 to 10.716) | –0.26 | 0.82 | 0 |
| Male, % | 10 | –0.003 (–0.172 to 0.166) | –0.07 | 0.95 | 0 |
| No. of cognitive measures | 10 | 0.065 (–0.768 to 0.900) | 0.34 | 0.77 | 0 |
| Quality of control group | 10 | –0.143 (–1.362 to 1.075) | –0.51 | 0.66 | 0 |

Categorical variables use a reference variable as recommended by CMA. R^2^ represents the total variance explained by the model when prior covariates are held constant.

CI, confidence interval; df, degrees of freedom.

# References

1. Naumann A, Bellebaum C, Daum I. Cognitive deficits in narcolepsy. *J Sleep Res.* 2006;**15**(3):329–338. doi: 10.1111/j.1365-2869.2006.00533.x.

2. Bayard S, Croisier Langenier M, Cochen De Cock V, Scholz S, Dauvilliers Y. Executive control of attention in narcolepsy. *PLoS One.* 2012;**7**(4):e33525. doi: 10.1371/journal.pone.0033525.

3. Ramm M, Jafarpour A, Boentert M, Lojewsky N, Young P, Heidbreder A. The Perception and Attention Functions test battery as a measure of neurocognitive impairment in patients with suspected central disorders of hypersomnolence. *J Sleep Res.* 2018;**27**(2):273–280. doi: 10.1111/jsr.12587.

4. Ha KS, Yoo HK, Lyoo IK, Jeong DU. Computerized assessment of cognitive impairment in narcoleptic patients. *Acta Neurol Scand.* 2007;**116**(5):312–316. doi: 10.1111/j.1600-0404.2007.00891.x.

5. Medrano-Martinez P, Peraita-Adrados R. Neuropsychological alterations in narcolepsy with cataplexy and the expression of cognitive deficits. *J Int Neuropsychol Soc.* 2020;**26**(6):587–595. doi: 10.1017/S1355617719001334.

6. Filardi M, Pizza F, Tonetti L, Antelmi E, Natale V, Plazzi G. Attention impairments and ADHD symptoms in adult narcoleptic patients with and without hypocretin deficiency. *PLoS One.* 2017;**12**(8):e0182085. doi: 10.1371/journal.pone.0182085.

7. Rieger M, Mayer G, Gauggel S. Attention deficits in patients with narcolepsy. *Sleep.* 2003;**26**(1):36–43.

8. Ramm M, Boentert M, Lojewsky N, Jafarpour A, Young P, Heidbreder A. Disease-specific attention impairment in disorders of chronic excessive daytime sleepiness. *Sleep Med.* 2019;**53**:133–140. doi: 10.1016/j.sleep.2018.09.021.

9. Schneider C, Fulda S, Schulz H. Daytime variation in performance and tiredness/sleepiness ratings in patients with insomnia, narcolepsy, sleep apnea and normal controls. *J Sleep Res.* 2004;**13**(4):373–383. doi: 10.1111/j.1365-2869.2004.00427.x.

10. Trotti LM, Saini P, Bremer E*, et al.* The Psychomotor Vigilance Test as a measure of alertness and sleep inertia in people with central disorders of hypersomnolence. *J Clin Sleep Med.* 2022;**18**(5):1395–1403. doi: 10.5664/jcsm.9884.

11. Thomann J, Baumann CR, Landolt HP, Werth E. Psychomotor vigilance task demonstrates impaired vigilance in disorders with excessive daytime sleepiness. *J Clin Sleep Med.* 2014;**10**(9):1019–1024. doi: 10.5664/jcsm.4042.

12. van Schie MK, Werth E, Lammers GJ, Overeem S, Baumann CR, Fronczek R. Improved vigilance after sodium oxybate treatment in narcolepsy: a comparison between in-field and in-laboratory measurements. *J Sleep Res.* 2016;**25**(4):486–496. doi: 10.1111/jsr.12386.

13. Huang YS, Hsiao IT, Liu FY*, et al.* Neurocognition, sleep, and PET findings in type 2 vs type 1 narcolepsy. *Neurology.* 2018;**90**(17):e1478–e1487. doi: 10.1212/WNL.0000000000005346.

14. Fronczek R, Middelkoop HA, van Dijk JG, Lammers GJ. Focusing on vigilance instead of sleepiness in the assessment of narcolepsy: high sensitivity of the Sustained Attention to Response Task (SART). *Sleep.* 2006;**29**(2):187–191.

15. Van Schie MK, Thijs RD, Fronczek R, Middelkoop HA, Lammers GJ, Van Dijk JG. Sustained attention to response task (SART) shows impaired vigilance in a spectrum of disorders of excessive daytime sleepiness. *J Sleep Res.* 2012;**21**(4):390–395. doi: 10.1111/j.1365-2869.2011.00979.x.

16. Gool JK, van der Werf YD, Lammers GJ, Fronczek R. The sustained attention to response task shows lower cingulo-opercular and frontoparietal activity in people with narcolepsy type 1: an fMRI study on the neural regulation of attention. *Brain Sci.* 2020;**10**(7):419. doi: 10.3390/brainsci10070419.

17. Janssens KAM, Quaedackers L, Lammers GJ*, et al.* Effect of treatment on cognitive and attention problems in children with narcolepsy type 1. *Sleep.* 2020;**43**(12):zsaa114. doi: 10.1093/sleep/zsaa114.

18. Zamarian L, Högl B, Delazer M*, et al.* Subjective deficits of attention, cognition and depression in patients with narcolepsy. *Sleep Med.* 2015;**16**(1):45–51. doi: 10.1016/j.sleep.2014.07.025.

19. Yoon SM, Joo EY, Kim JY, Hwang KJ, Hong SB. Is high IQ protective against cognitive dysfunction in narcoleptic patients? *J Clin Neurol.* 2013;**9**(2):118–124. doi: 10.3988/jcn.2013.9.2.118.

20. Witt ST, Drissi NM, Tapper S*, et al.* Evidence for cognitive resource imbalance in adolescents with narcolepsy. *Brain Imaging Behav.* 2018;**12**(2):411–424. doi: 10.1007/s11682-017-9706-y.

21. Delazer M, Hogl B, Zamarian L*, et al.* Executive functions, information sampling, and decision making in narcolepsy with cataplexy. *Neuropsychology.* 2011;**25**(4):477–487. doi: 10.1037/a0022357.

22. Moraes M, Rossini S, Reimao R. Executive attention and working memory in narcoleptic outpatients. *Arq Neuropsiquiatr.* 2012;**70**(5):335–340. doi: 10.1590/s0004-282x2012005000007.

23. Kim H, Suh S, Joo EY, Hong SB. Morphological alterations in amygdalo-hippocampal substructures in narcolepsy patients with cataplexy. *Brain Imaging Behav.* 2016;**10**(4):984–994. doi: 10.1007/s11682-015-9450-0.

24. van Holst RJ, Janssen LK, van Mierlo P*, et al.* Enhanced food-related responses in the ventral medial prefrontal cortex in narcolepsy type 1. *Sci Rep.* 2018;**8**(1):16391. doi: 10.1038/s41598-018-34647-6.

25. Mazzetti M, Campi C, Mattarozzi K*, et al.* Semantic priming effect during REM-sleep inertia in patients with narcolepsy. *Brain Res Bull.* 2006;**71**(1–3):270–278. doi: 10.1016/j.brainresbull.2006.09.011.

26. Mazzetti M, Bellucci C, Mattarozzi K, Plazzi G, Tuozzi G, Cipolli C. REM-dreams recall in patients with narcolepsy-cataplexy. *Brain Res Bull.* 2010;**81**(1):133–140. doi: 10.1016/j.brainresbull.2009.10.021.

27. Mazzetti M, Plazzi G, Campi C*, et al.* Sleep-dependent consolidation of motor skills in patients with narcolepsy-cataplexy. *Arch Ital Biol.* 2012;**150**(2–3):185–193. doi: 10.4449/aib.v150i2/3.1412.

28. Park YK, Kwon OH, Joo EY*, et al.* White matter alterations in narcolepsy patients with cataplexy: tract-based spatial statistics. *J Sleep Res.* 2016;**25**(2):181–189. doi: 10.1111/jsr.12366.

29. Bayard S, Abril B, Yu H, Scholz S, Carlander B, Dauvilliers Y. Decision making in narcolepsy with cataplexy. *Sleep.* 2011;**34**(1):99–104. doi: 10.1093/sleep/34.1.99.

30. Bayard S, Langenier MC, Dauvilliers Y. Effect of psychostimulants on impulsivity and risk taking in narcolepsy with cataplexy. *Sleep.* 2013;**36**(9):1335–1340. doi: 10.5665/sleep.2958.

31. Dimitrova A, Fronczek R, Van der Ploeg J*, et al.* Reward-seeking behavior in human narcolepsy. *J Clin Sleep Med.* 2011;**7**(3):293–300. doi: 10.5664/JCSM.1076.

32. Cipolli C, Campana G, Campi C*, et al.* Sleep and time course of consolidation of visual discrimination skills in patients with narcolepsy-cataplexy. *J Sleep Res.* 2009;**18**(2):209–220. doi: 10.1111/j.1365-2869.2008.00712.x.

33. Cipolli C, Bellucci C, Mattarozzi K, Mazzetti M, Tuozzi G, Plazzi G. Story-like organization of REM-dreams in patients with narcolepsy-cataplexy. *Brain Res Bull.* 2008;**77**(4):206–213. doi: 10.1016/j.brainresbull.2008.07.012.

34. Joo EY, Kim SH, Kim ST, Hong SB. Hippocampal volume and memory in narcoleptics with cataplexy. *Sleep Med.* 2012;**13**(4):396–401. doi: 10.1016/j.sleep.2011.09.017.

35. Saletu M, Anderer P, Saletu-Zyhlarz GM, Mandl M, Zeitlhofer J, Saletu B. Event-related-potential low-resolution brain electromagnetic tomography (ERP-LORETA) suggests decreased energetic resources for cognitive processing in narcolepsy. *Clin Neurophysiol.* 2008;**119**(8):1782–1794. doi: 10.1016/j.clinph.2008.04.297.

36. Asp A, Lund F, Benedict C, Wasling P. Impaired procedural memory in narcolepsy type 1. *Acta Neurol Scand.* 2022;**146**(2):186–193. doi: 10.1111/ane.13651.

37. Marcotte TD, Scott JC, Kamat R, Heaton RK. Neuropsychology and the prediction of everyday functioning. In: Marcotte TD, Grant I, eds. Neuropsychology of everyday functioning. New York, NY: The Guildford Press; 2010: 5–38.

38. Scolari M, Seidl-Rathkopf KN, Kastner S. Functions of the human frontoparietal attention network: Evidence from neuroimaging. *Curr Opin Behav Sci.* 2015;**1**:32–39. doi: 10.1016/j.cobeha.2014.08.003.
